# A novel *O*-linked glycan modulates *Campylobacter jejuni* major outer membrane protein-mediated adhesion to human histo-blood group antigens and chicken colonization

**DOI:** 10.1098/rsob.130202

**Published:** 2014-01-22

**Authors:** Jafar Mahdavi, Necmettin Pirinccioglu, Neil J. Oldfield, Elisabet Carlsohn, Jeroen Stoof, Akhmed Aslam, Tim Self, Shaun A. Cawthraw, Liljana Petrovska, Natalie Colborne, Carina Sihlbom, Thomas Borén, Karl G. Wooldridge, Dlawer A. A. Ala'Aldeen

**Affiliations:** 1 School of Life Sciences, University of Nottingham, Nottingham NG7 2RD, UK; 2 Department of Chemistry, University of Dicle, Diyarbakir 21280, Turkey; 3 Proteomics Core Facility, Sahlgrenska Academy, University of Gothenburg, PO Box 413, 405 30 Gothenburg, Sweden; 4 Animal Health and Veterinary Laboratories Agency, Weybridge, Surrey KT15 3NB, UK; 5 Department of Medical Biochemistry and Biophysics, Umeå University, Umeå 901 87, Sweden

**Keywords:** *Campylobacter jejuni*, histo-blood group antigens, FlaA, major outer membrane protein, *O*-glycosylation, biofilm

## Abstract

*Campylobacter jejuni* is an important cause of human foodborne gastroenteritis; strategies to prevent infection are hampered by a poor understanding of the complex interactions between host and pathogen. Previous work showed that *C. jejuni* could bind human histo-blood group antigens (BgAgs) *in vitro* and that BgAgs could inhibit the binding of *C. jejuni* to human intestinal mucosa *ex vivo.* Here, the major flagella subunit protein (FlaA) and the major outer membrane protein (MOMP) were identified as BgAg-binding adhesins in *C. jejuni* NCTC11168*.* Significantly, the MOMP was shown to be *O-*glycosylated at Thr^268^; previously only flagellin proteins were known to be *O-*glycosylated in *C. jejuni*. Substitution of MOMP Thr^268^ led to significantly reduced binding to BgAgs. The *O-*glycan moiety was characterized as Gal(β1–3)-GalNAc(β1–4)-GalNAc(β1–4)-GalNAcα1-Thr^268^; modelling suggested that *O-*glycosylation has a notable effect on the conformation of MOMP and this modulates BgAg-binding capacity. Glycosylation of MOMP at Thr^268^ promoted cell-to-cell binding, biofilm formation and adhesion to Caco-2 cells, and was required for the optimal colonization of chickens by *C. jejuni*, confirming the significance of this *O-*glycosylation in pathogenesis.

## 2. Introduction

*Campylobacter jejuni* is the most common causative agent of bacterial gastroenteritis reported worldwide [1]. It is responsible for a spectrum of intestinal symptoms, including watery diarrhoea, bloody diarrhoea, vomiting, fever and abdominal cramps. Symptoms range from mild to severe and may lead to death in immunocompromised patients [2]. Acute-phase disease is sometimes followed by serious long-term sequelae, including reactive arthritis [3,4], the autoimmune neuropathies Guillain–Barré syndrome (GBS) and Miller–Fisher syndrome [5] and immunoproliferative small intestinal disease [6]. The biochemical structures of gangliosides play a crucial role in the pathogenesis of GBS. Infection with *C. jejuni* is responsible for 35% of all types of GBS and 20–30% of GBS-linked antibodies are directed against gangliosides [7].

Prevention and treatment of the primary and secondary consequences of *Campylobacter* infection are hampered by a poor understanding of the detailed molecular interplay between cells of the gastrointestinal lining and the pathogen. Following ingestion, *C. jejuni* infect and invade the epithelium of the small intestine and colon. Infection is dependent on motility mediated by polar flagella and outer membrane adhesins, including PEB1a, PEB3, JlpA, MOMP, CapA and CadF [8–13]. Thus, surface-exposed bacterial ligands play major roles in mediating mucosal adhesion and invasion. *Campylobacter jejuni* harbours both *N*- and *O*-linked glycosylation systems [14,15]; both have been implicated in adhesion to, and invasion of, epithelial cells *in vitro* [16] and host colonization and virulence *in vivo* [17,18]. The only target of the *O*-linked glycosylation system described to date is flagellin [19], whereas the *N-*linked system has a variety of targets, including periplasmic and outer membrane proteins [20].

Ruiz-Palacios and co-workers [21,22] showed that fucosylated sugar components in human breast milk could inhibit *C. jejuni* binding to HEp-2 cells *in vitro* and to human intestinal mucosa *ex vivo*, and also prevent *C. jejuni* infection of mice *in vivo*. Subsequent solid-phase assays confirmed the binding of *C. jejuni* strains, grown under host-like conditions, to a broad range of fucose (and galactose)-containing glycans, with different strains exhibiting differing binding profiles and avidities [21,23]; however, the identity of the *C. jejuni* lectin(s) responsible for this binding has not to date been determined. The common ABO histo-blood group antigens (BgAgs) comprise a complex and polymorphic group of fucosylated carbohydrates expressed on the surfaces of erythrocytes, but they are also highly expressed in the oro-gastro-intestinal (OGI) epithelium, as well as endothelial cells, and in secretions, such as milk, saliva and tears [24,25]. Their common denominators are the types I and II glycan core chains which may be fucosylated in the bone marrow by the H-II-(fucosyl)-transferase, before being absorbed into the surface of erythrocytes [26] or by the H-I-(fucosyl)-transferase in the mucosal glandular tissues, for example that along the OGI tract. Fucosylated glycans are substrates for further glycosylation reactions that give rise to the epitopes defining the A, B and Lewis BgAgs.

The ABO (or ABH) and Lewis BgAgs are epidemiologically associated with susceptibility to several infectious agents [26]. Among enteropathogenic bacteria, *Helicobacter pylori* bind the H-I and Le^b^ antigens [27]. The *H. pylori* BabA (blood group antigen-binding) and SabA (sialic acid-binding adhesin) proteins are the best described ABO/Le^b^ and sialyl-Lewis x (sLe^x^) antigen-binding adhesins and predictors for the development of overt gastric disease [28,29].

Here, we identify the *C. jejuni* surface proteins FlaA and MOMP as BgAg-binding adhesins. Furthermore, we show that flagellins are not the only *O-*glycosylated proteins in *C. jejuni*; MOMP is also *O-*glycosylated, a modification that influences the MOMP conformation and thus BgAg interactions. MOMP *O-*glycosylation promoted biofilm formation and auto-aggregation, and was required for optimal adhesion to Caco-2 cells and colonization of chickens, confirming the significance of this post-translational modification in *C. jejuni* pathogenesis.

## 3. Results

### 3.1. *Campylobacter jejuni* binds a wide range of human histo-BgAgs

**Figure 1.**
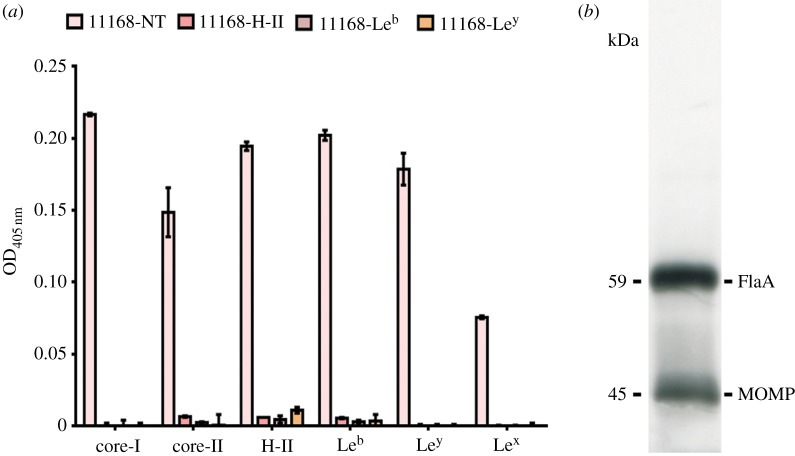
Binding of *C. jejuni* to BgAgs and identification of *C. jejuni* BgAg-binding adhesins. (*a*) Binding of digoxigenin (DIG)-labelled *C. jejuni* NCTC11168 to ELISA wells coated with BgAgs. Specific binding was calculated by subtracting the bovine serum albumin (BSA; negative control) values from the BSA–BgAg absorbance. Binding was significantly inhibited (*p* < 0.05) by the pre-incubation of bacteria with soluble glycoconjugates H-II, Le^b^ or Le^y^ prior to adding to the ELISA plate. NT, non-treated. Error bars: mean of triplicate values ± s.e.m. from three independent experiments. (*b*) Identification of BgAg-binding proteins from *C. jejuni* NCTC11168 using re-tagging (baits used: H-II; H-I and Le^b^). Two proteins were identified at sizes of 45 and 59 kDa, corresponding to MOMP and FlaA, respectively.

To confirm that *C. jejuni* binds BgAgs, the core-I, core-II, H-II, Le^b^, Le^y^ and Le^x^ BgAgs were adsorbed to the surface of amino-linked ELISA plates and incubated with digoxigenin (DIG)-labelled *C. jejuni* strain NCTC11168. The structures of these molecules are shown in the electronic supplementary material, table S1. NCTC11168 bound to each of the glycoconjugates tested (figure 1*a*). Importantly, the pre-incubation of NCTC11168 with soluble BgAgs inhibited binding in this assay (figure 1*a*). In addition, 45 clinical isolates of *C. jejuni* (see electronic supplementary material, table S2) also bound to many of the same BgAgs, albeit to a varied degree (see electronic supplementary material, table S3) confirming that BgAg binding is a widespread phenotypic trait among *C. jejuni* isolates.

### 3.2. *Campylobacter jejuni* FlaA and MOMP mediate binding to human BgAgs

**Figure 2.**
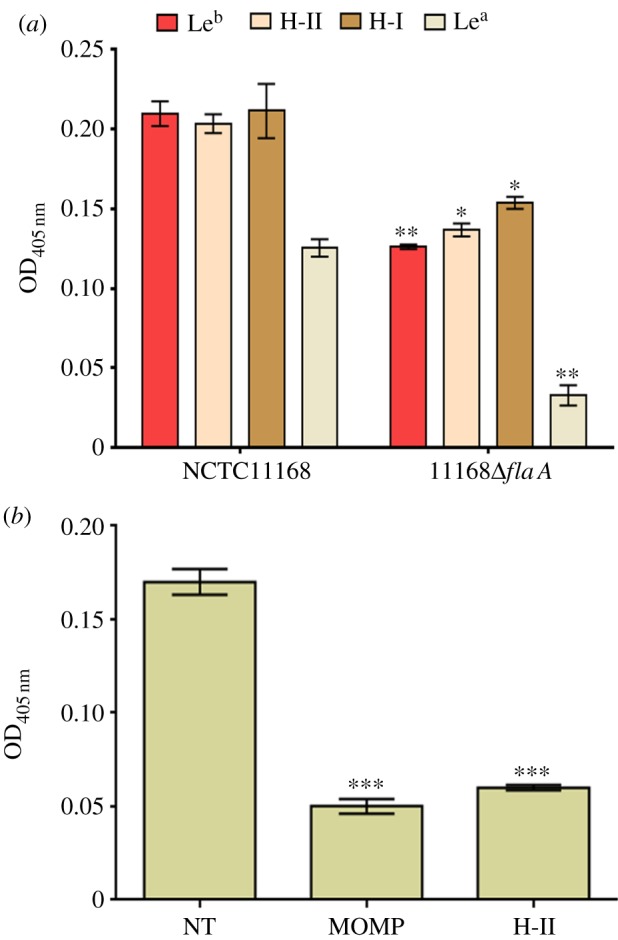
FlaA and MOMP are required for *C. jejuni* BgAg binding. (*a*) NCTC11168 and its Δ*flaA* mutant derivative were examined for binding to Le^b^, H-II, H-I and Le^a^. Binding of 11168Δ*flaA* was significantly reduced compared with the wild-type (*p < 0.05; **p < 0.01). Error bars: mean of triplicate values ± s.e.m. from three independent experiments. (*b*) Binding of NCTC11168 to H-II glycoconjugate in the absence of inhibitor (non-treated, NT) or after H-II coated plates were treated with purified NCTC11168 MOMP before the addition of bacteria or after NCTC11168 were pre-incubated with H-II glycoconjugate (H-II). ***significantly reduced compared with non-treated (*p* < 0.001). Error bars: mean of triplicate values ± s.e.m. from three independent experiments.

A technique employing a multi-functional cross-linker, termed as retagging [29], was used for the identification of *C. jejuni* BgAg-binding adhesins. NCTC11168 cells were incubated with BgAg conjugated to a light-activated cross-linker. Following photo-activation and subsequent exposure to reducing conditions to allow transfer of the reactive biotin moiety to molecules in close proximity to the BgAgs, bacteria were lysed and the biotin-tagged *C. jejuni* proteins purified with streptavidin-coated magnetic beads, as previously described [29]. Extracted biotin-tagged proteins were separated by SDS-PAGE and identified using MALDI-TOF. Two proteins were identified: the 45 kDa major outer membrane protein (MOMP) and the 59 kDa flagellin FlaA (figure 1*b*). FlaA is the major flagellin and is required for the expression of functional flagella. To confirm that this molecule contributed to the observed BgAg-binding activity, *flaA* was deleted in NCTC11168 and the mutant tested for its ability to bind to BgAgs in ELISA experiments. Deletion of *flaA* led to significantly reduced binding of the NCTC11168 mutant to all examined BgAgs (figure 2*a*). MOMP, encoded by *porA*, is an essential multi-functional porin [30]. As it was not possible to delete *porA*, we purified the MOMP under non-denaturing conditions from strain NCTC11168. The purified protein significantly inhibited binding of NCTC11168 to the H-II glycoconjugate (figure 2*b*).

### 3.3. NCTC11168 MOMP is *O-*glycosylated

**Figure 3.**
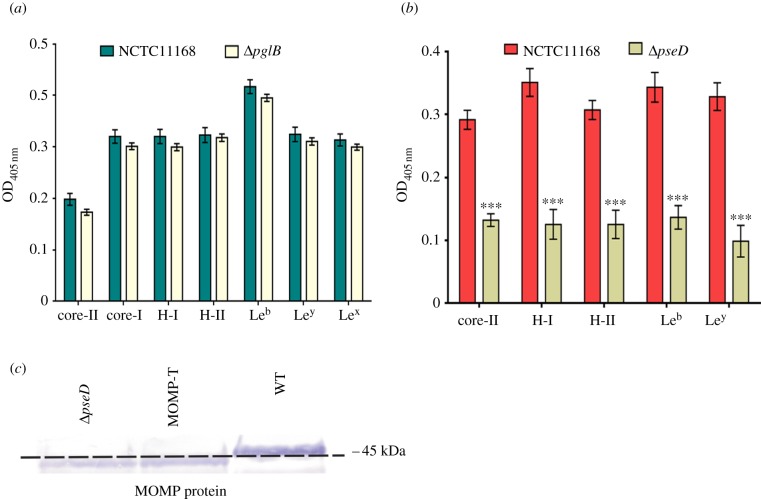
*O*-Glycosylation but not *N-*glycosylation modulates *C. jejuni* BgAg binding. (*a*) NCTC11168 and 11168Δ*pglB* were examined for binding to core-II, core-I, H-I, H-II, Le^b^, Le^x^ and Le^y^. Binding was not significantly reduced following *pglB* mutation. Error bars: mean of triplicate values ± s.e.m. from three independent experiments. (*b*) NCTC11168 and 11168Δ*pseD* were examined for binding to core-II, H-I, H-II, Le^b^ and Le^y^. The binding of 11168Δ*pseD* was significantly reduced compared with the wild-type (***p < 0.001). Error bars: mean of triplicate values ± s.e.m. from three independent experiments. (*c*) Immunoblot analysis of purified MOMP from wild-type NCTC11168, 11168MOMP^268T/G^ and 11168Δ*pseD* strains using anti-MOMP antibodies. MOMP from the latter two strains showed an apparent size shift compared with the wild-type protein indicating a loss of glycosylation.

Both *O-* and *N-*glycosylation systems have been described in *C. jejuni.* FlaA is a known glycoprotein that is post-translationally modified with *O*-linked glycans that can constitute up to 10% of the molecule's mass [31]. Furthermore, an increasing number of periplasmic and outer membrane proteins of *C. jejuni* have been found to be *N-*glycosylated. Inhibition of key enzymatic steps in each pathway blocks the addition of glycans to target proteins. In the *N-*glycosylation (Pgl) pathway, the oligosaccharyltransferase PglB is critical for transfer of the final glycan to the target protein in the periplasm. Deletion of *pglB* had no detectable impact on bacterial binding to any of the examined BgAgs (figure 3*a*), indicating that the *N-*glycosylation system does not influence BgAg binding.

**Figure 4.**
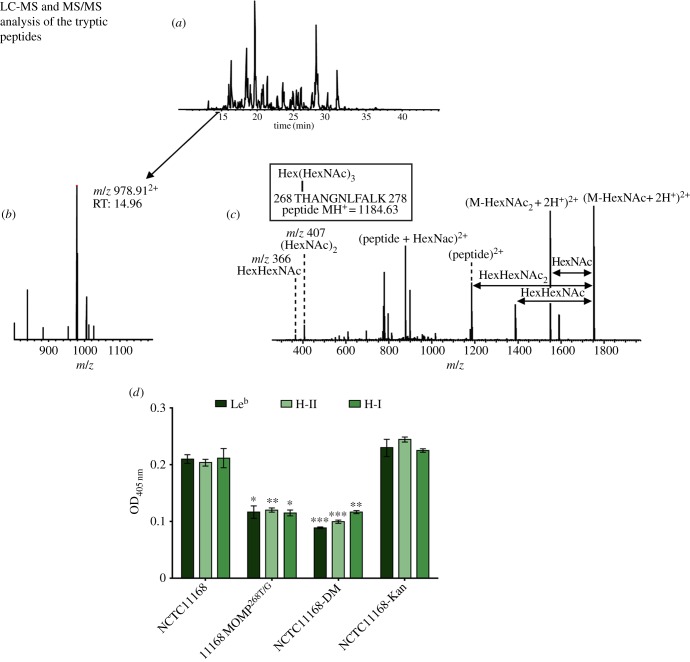
Overview of the LC-MS/MS analysis of NCTC11168 MOMP and confirmation of the role of Thr^268^ glycosylation in BgAg binding. (*a*) Base peak chromatogram: tryptic peptides are loaded on an online coupled C18 column and eluted into the mass spectrometer for analysis. (*b*) MS precursor scan of the double charged glycosylated peptide at *m*/*z* 978.91. (*c*) CID-MS/MS spectrum of the selected ion. (*d*) Substitution of the glycosylation site in NCTC11168 MOMP (Thr^268^ to Gly) was achieved and the binding to Le^b^, H-II and H-I was examined. Also included was a double mutant (DM) in which *flaA* was also inactivated and 11168-Kan, which contains the resistance cassette at the *porA* locus but without the substitution. Compared with wild-type, BgAg binding was significantly reduced in both 11168MOMP^268T/G^ and 11168-DM strains. 11168-Kan showed no significant difference in BgAg-binding activity compared with NCTC11168. Error bars: mean of triplicate values ± s.e.m. from three independent experiments. *p < 0.05; **p < 0.01; ***p < 0.001.

The predominant *O*-glycans attached to the *Campylobacter* flagellum are derivatives of pseudaminic acid (Pse) or legionaminic acid (Leg), which are C9 sugars related to sialic acids [32]. Mutation of *pseD*, which encodes a putative PseAm transferase, results in a fully motile phenotype [19,33], but with a loss of PseAm from the flagellin [19]. Mutation of *pseD* resulted in a significant reduction in the binding of NCTC11168 to all examined BgAgs (figure 3*b*), suggesting that *O-*glycosylation of BgAg ligands is required for optimal BgAg binding. Intriguingly, when MOMPs purified from NCTC11168 and 11168Δ*pseD* were compared by immunoblotting, the latter exhibited an apparent size shift compared with MOMP from the wild-type strain, consistent with the loss of glycan modifications (figure 3*c*) and reminiscent of the size shift seen when comparing glycosylated and unglycosylated flagellin proteins [31]. We therefore hypothesized that, in addition to flagellin proteins, MOMP may also be *O-*glycosylated. To address this, MOMP from NCTC11168 was analysed by Nanoflow LC-MS/MS FT/ICR following in-gel protein digestion as described by Shevchenko *et al.* [34]. The MS/MS analysis showed that a peptide corresponding to amino acids 268–278 of NCTC11168 MOMP was glycosylated; specifically, the amino acid Thr^268^ was carrying Hex-(Hex*N*-acetylamine)_3_ (where Hex can be glucose or galactose; figure 4*a–c*). To confirm that Thr^268^ was indeed the amino acid residue targeted by the *O*-glycosylation system, we substituted Gly for Thr^268^ yielding a MOMP^268T/G^ derivative in NCTC11168. This substitution had no deleterious effect on *in vitro* growth (data not shown), but the apparent size of the 11168 MOMP^268T/G^ protein was distinct from that of wild-type MOMP and indistinguishable from that of MOMP purified from 11168Δ*pseD* (figure 3*c*), confirming that Thr^268^ was glycosylated in the wild-type protein, the glycosylation was responsible for the altered electrophoretic mobility and that PseD was required for this modification. Nanoflow LC-MS/MS FT/ICR analysis of purified MOMP^268T/G^ confirmed the absence of the Hex-(Hex*N*-acetylamine)_3_ glycosylation.

### 3.4. *O-*Glycosylation of NCTC11168 MOMP effects BgAg binding

The ability of the MOMP^268T/G^ mutant to bind to a range of BgAgs in ELISA assays was examined, along with a double mutant strain in which *flaA* had also been inactivated. The MOMP^268T/G^ substitution significantly reduced BgAg binding (figure 4*d*). To rule out polar effects linked to the insertion of a kanamycin resistance cassette at the end of *porA* (used to facilitate selection for the MOMP^268T/G^ substitution), we also constructed 11168-Kan, which contains the resistance cassette at the *porA* locus but without the substitution. This strain showed no significant difference in BgAg-binding activity compared with NCTC11168 (figure 4*d*).

### 3.5. NCTC11168 MOMP glycosylation is an *O*-linked trimeric form of T-antigen

**Figure 5.**
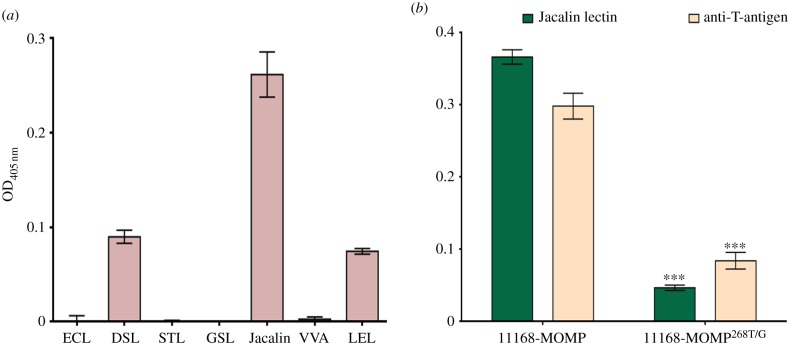
Detection of glycan constituents of purified MOMP from NCTC11168 using biotinylated labelled lectins and antibodies against glycans. (*a*) Binding of the following glycans: GSL II, *Griffonia* (*Bandeiraea*) *simplicifolia* lectin II; DSL, *Datura Stramonium* lectin; ECL, *Erythrina cristagalli* lectin; LEL, *Lycopersicon esculentum* (tomato) lectin; STL, *Solanum tuberosum* (potato) lectin; VVA, *Vicia villosa* agglutinin; and Jacalin, *Artocarpus integrifolia* lectin to immobilized NCTC11168 MOMP was investigated using ELISA. Jacalin lectin showed the greatest degree of binding to NCTC11168 MOMP. Jacalin lectin specifically recognizes Gal(β1–3)-GalNAcα1-Ser/Thr (T-antigen). (*b*) ELISA confirmation using anti-T-antigen monoclonal antibody or Jacalin lectin to confirm that the glycan detected on NCTC11168 MOMP was T-antigen. 11168MOMP^268T/G^ showed significantly reduced binding to either Jacalin lectin or anti-T antigen compared with NCTC11168 MOMP. Error bars: mean of triplicate values ± s.e.m. from three independent experiments. ***p < 0.001.

Lectin binding was used for further characterization of the *O-*glycan attached to NCTC11168 MOMP. Seven different lectins with overlapping specificities (see electronic supplementary material, table S4) were tested. Purified NCTC11168 MOMP showed significant binding to the lectins Jacalin [35] and, to a lesser extent, DSL and LEL (figure 5*a*). Among the galactose-specific lectins, Jacalin exhibits specificity for human tumour-specific Thomsen-Friedenreich disaccharide (T-antigen; Gal(β1–3)-GalNAcα1-Ser/Thr) [36]. To confirm that the Jacalin binding was the result of *O-*glycosylation of amino acid Thr^268^, MOMPs purified from NCTC11168 and NCTC11168 MOMP^268T/G^ were probed with monoclonal anti-T-antigen in ELISA assays (figure 5*b*). Both the antibody and Jacalin lectin bound strongly to MOMP purified from NCTC11168; significant reduced binding was observed to MOMP^268T/G^ (figure 5*b*). Combined with the previous MS/MS analysis suggesting a Hex-(Hex*N*-acetylamine)_3_ glycan, our lectin-binding analysis indicates that the MOMP glycan in NCTC11168 is likely to be the *O*-linked trimeric form of T-antigen: Gal(β1–3)-GalNAc(β1–4)-GalNAc(β1–4)-GalNAcα1-Thr^268^ (electronic supplementary material, figure S1).

### 3.6. Modelling the structure of glycosylated and non-glycosylated NCTC11168 MOMP

**Figure 6.**
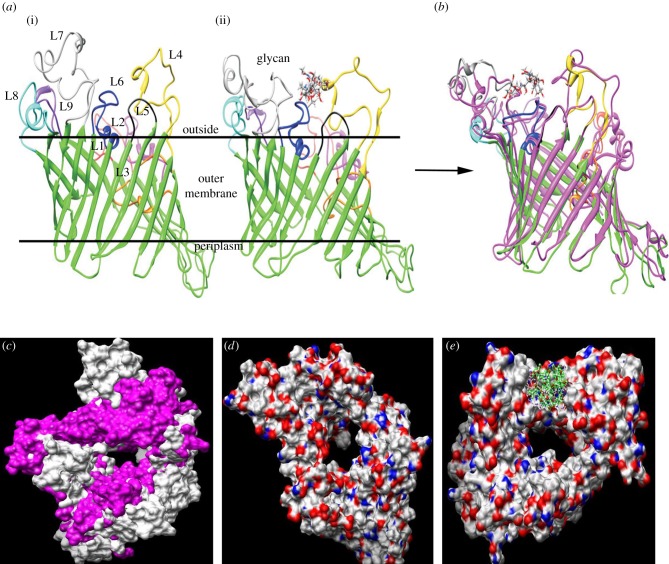
Three-dimensional model of MOMP. (*a*) Non-glycosylated (i) or glycosylated (ii) at Thr^268^ (loop 6) of NCTC11168 MOMP embedded in the outer membrane. Approximate boundaries of the hydrophobic regions of the proteins predicted to be embedded in the outer membrane are represented by horizontal lines. (*b*) The superimposed lowest energy structure of non-glycosylated MOMP on the lowest energy structure of glycosylated MOMP (magenta) with RMSD of 1.291. Loops are shown in the following colours: β strands are green; L1 (residues 41–60), red; L2 (residues 87–109), magenta; L3 (residues 128–147), orange; L4 (residues 169–200), yellow; L5 (residues 227–233), black; L6 (residues 256–274), blue; L7 (residues 296–333), grey; L8 (residues 360–379), cyan; L9 (residues 399–414), purple. (*c*) Superimposed surface representation of the structures of non-glycosylated MOMP (grey) on its glycosylated form (magenta) viewed from the external face. Surface representation of the structures of non-glycosylated MOMP (*d*) and its glycosylated form (*e*), viewed from the external face.

Although Gram-negative bacterial porins show considerable diversity in their primary sequences, they are remarkably similar in their secondary and tertiary structure, in which beta strands combine to form a trans-membrane beta-barrel structure [37]. CD spectroscopy analysis demonstrated that the folded MOMP monomer mainly comprised *β*-sheet secondary structure [38]. Accordingly, two-dimensional crystallographic analysis showed that MOMP is structurally related to the family of trimeric bacterial porins [39]. Here, we utilized the beta-barrel structure of the anion-selective porins of *Comamonas acidovorans* (1E54.pdb) and *Pseudomonas aeruginosa* (2QTK.pdb) as templates to construct a model of *C. jejuni* NCTC11168 MOMP. The lowest energy structures of glycosylated and non-glycosylated MOMP at 300 K were calculated; these comprised nine loops and 18 beta strands (figure 6*a*). The structures of non-glycosylated MOMP and MOMP glycosylated at residue 268 were calculated and superimposed (figure 6*b*,*c*); the modification was predicted to result in major conformational changes affecting loops 4, 6 and 7 (corresponding to amino acids 169–200, 256–274 and 296–333, respectively); thus we hypothesized that the *O-*glycosylation of MOMP leads to conformation changes in the molecule which might favour BgAg binding. In addition, further simulations showed that the protein has a canal-like cavity, which is expected to be capable of accommodating very large molecules. However, non-glycosylated MOMP has a larger cavity compared with its glycosylated form (figure 6*d*,*e*).

### 3.7. Modelling NCTC11168 MOMP–BgAg interactions

**Figure 7.**
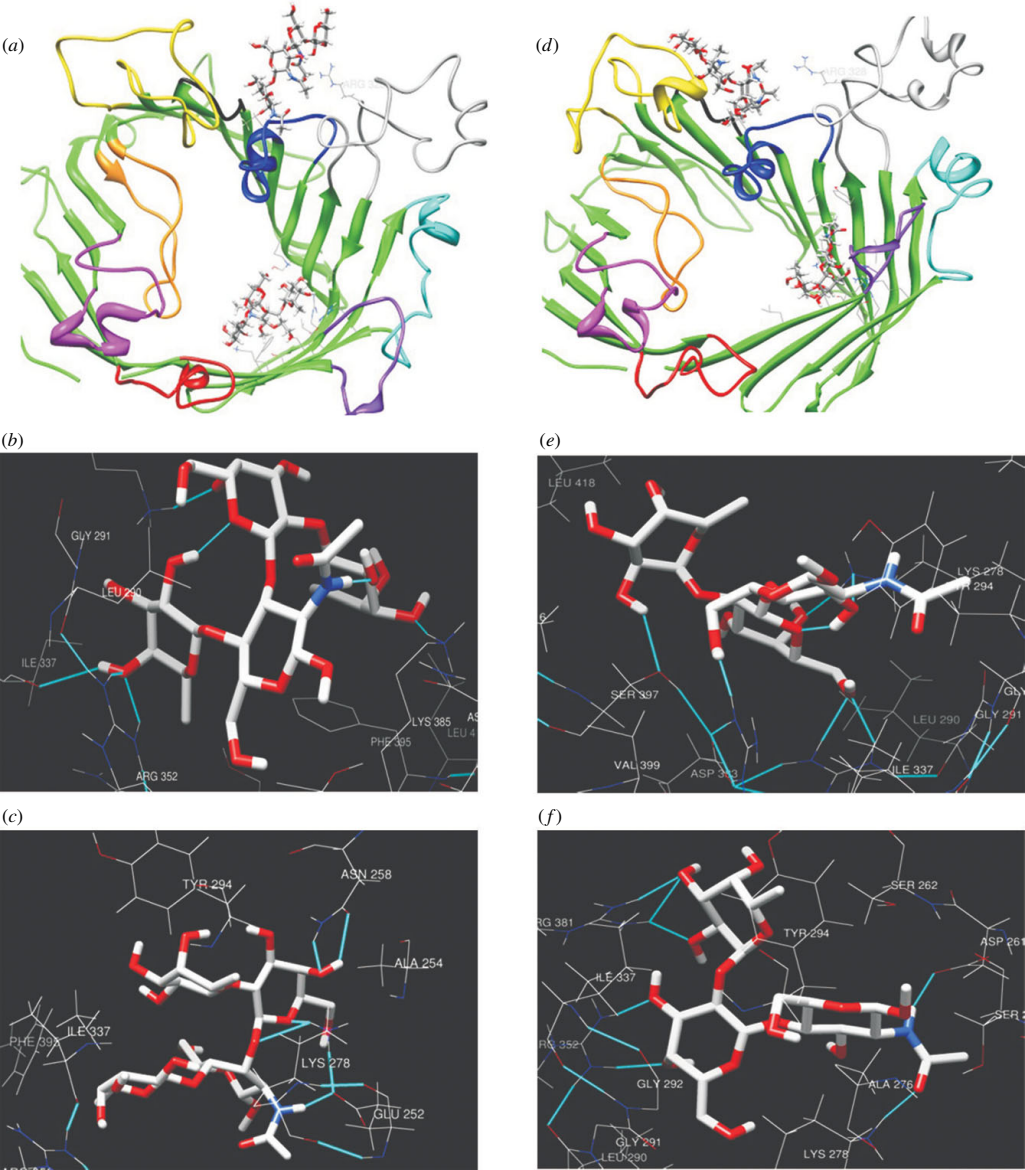
Influence of glycosylation on conformational changes induced by ligand binding. A stereo cartoon of the MOMP backbone viewed from the extracellular side is presented. Regions are shown in the following colours: green (β strands); red (L1); magenta (L2); orange (L3); yellow (L4); black (L5); blue (L6); grey (L7); cyan (L8); purple (L9). The complexes are with Le^b^ (*a*) and H-II (*d*)*.* In addition, hydrogen bonds shown in light blue correspond to amino acids residues, which are putatively involved in the interactions of glycosylated MOMP (*b* and *e*) and its non-glycosylated form (*c* and *f*) with Le^b^ and H-II, respectively, in their active sites.

To further support our hypothesis, Le^b^ (as an example of a type-1 Lewis antigen) and H-II (as an example of a type-2 antigen; electronic supplementary material, figure S2) were docked into the central channel-forming cavity of NCTC11168 MOMP predicted in the model structures of glycosylated MOMP (figure 7*a* and *d*, respectively) and the complexes computed in molecular dynamics (MD) [9] simulations. Interestingly, the predicted interaction between the galactose residues with Arg^328^ is broken in the complex of MOMP and H-II, in which the region containing the glycosylated residue moves towards loop 4 resulting in a new interaction with the residues Thr^186^ and Thr^187^. A similar conformational change was not predicted when the interaction between glycosylated MOMP and Le^b^ was modelled. The average energies derived from MD simulations of complexes are listed in the electronic supplementary material, table S5. The introduction of the ligands leads to conformational changes affecting the longest of the predicted loops (loops 4 and 7) in particular. It was found that the predicted glycosylated MOMP structure was relatively stable compared with the non-glycosylated form, with only loop 7 predicted to undergo marked conformational changes when glycosylated MOMP is complexed with BgAgs. This prediction suggests that glycosylation enhances the stability of the protein.

Figure 7 highlights the residues of glycosylated and non-glycosylated MOMP contributing to the interaction of these proteins with Le^b^ (*b* and *c*, respectively) and H-II (*e* and *f*, respectively). It is apparent that the glycosylated MOMP has favourable interactions with Le^b^ compared with the unmodified version. Specifically, the residues Arg^352^, Lys^278^ and Lys^385^ are predicted to be major contributors to the interaction of the glycosylated protein with Le^b^ via hydrogen bonds, whereas only the residues Asn^258^ and Lys^278^ of non-glycosylated MOMP are predicted to interact with Le^b^. Residues Arg^352^ and Lys^385^ are both within loop 7, which is predicted to be more susceptible to conformation changes as a result of glycosylation or interaction with BgAgs in MD simulations (figure 7*b*). Incorporation of the glycosyl group with this loop thus appears to stabilize a conformation that favours interaction with Le^b^.

Calculations also show that the glycosylated protein has more favourable van der Waals interactions compared with the non-glycosylated protein: the residues Leu^290^, Tyr^294^, Phe^395^ and Ile^337^ are located over the hydrophobic surface of Le^b^ in the complex of glycosylated MOMP but not in the complex with non-glycosylated MOMP (figure 7*b* and *c*, respectively). This is reflected in a 67 kcal mol^−1^ van der Waals energy difference between the two complexes. H-II is predicted to be bound to both versions of the protein in a similar manner to Le^b^. The residues Lys^278^, Arg^352^ and Arg^381^ are involved in the complex of both glycosylated and non-glycosylated MOMP with H-II (figure 7*e* and *f*, respectively). The two complexes differ in that residue Asp^261^ contributes to the interaction of H-II with non-glycosylated MOMP, but not glycosylated MOMP, whereas the reverse is true for Ser^397^. MD calculations also show that active sites of the glycosylated and non-glycosylated proteins have different conformational orientations with respect to the two ligands.

We attempted to substitute five amino acids (S262-G, K278-G, R352-G, R381-G and S397-Y) within the predicted binding site of NCTC11168 MOMP (singly or in various combinations) but were unable to do so, suggesting perhaps that these amino acid residues are important for MOMP stability and thus function. Overall, our modelling confirms that the *O-*glycosylation of MOMP favours BgAg binding.

### 3.8. *O-*Glycosylation of NCTC11168 MOMP effects auto-agglutination, biofilm formation, adherence to Caco-2 cells and chicken colonization

**Figure 8.**
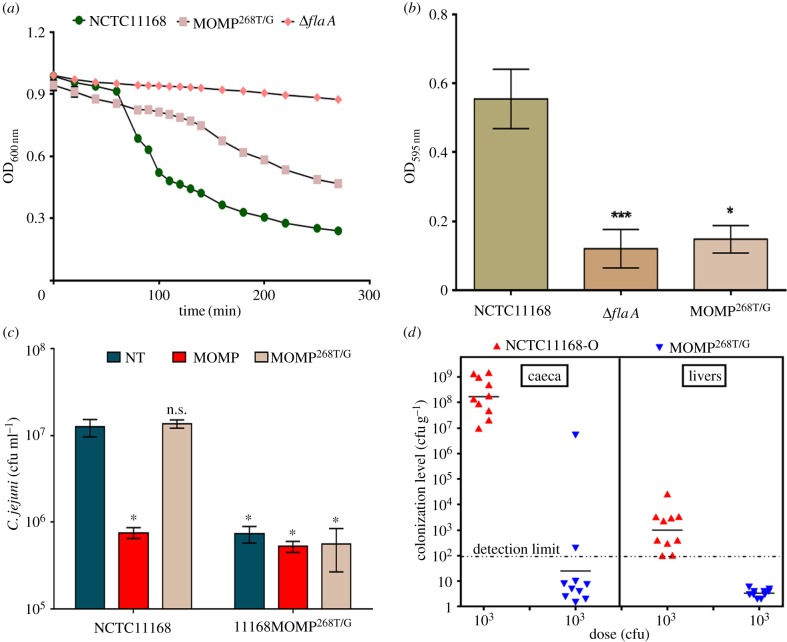
*O-*glycosylation of MOMP effects auto-agglutination, biofilm formation, adherence to Caco-2 cells and chicken colonization. (*a*) The ability of 11168MOMP^268T/G^ to auto-agglutination was significantly reduced compared with wild-type 11168 (*p* < 0.0001). 11168Δ*flaA* was used as a negative control for aggregation. Error bars (too small to be seen) = mean of triplicate values on three occasions ± s.e.m. (*b*) Similarly, the ability of 11168MOMP^268T/G^ to form biofilms was significantly reduced compared with wild-type NCTC11168. 11168Δ*flaA* was used as a negative control for biofilm formation. Error bars = mean of triplicate values on three occasions ± s.e.m. *p < 0.05; ***p < 0.001. (*c*) Binding of NCTC11168 and 11168MOMP^268T/G^ to Caco-2 cells in the absence of inhibitor (non-treated, NT) or following pre-treatment of Caco-2 cells with purified MOMP or MOMP^268T/G^. *Significantly reduced compared with the binding of non-treated NCTC11168 (*p* < 0.05). Error bars: mean of triplicate values ± s.e.m. from three independent experiments. (*d*) Colonization of chickens by NCTC11168-O and 11168-O MOMP^268T/G^ mutant 7 days post-infection. The geometric mean colonization levels in caeca are significantly reduced (*p* < 0.0001) and are below the limit of detection (10^2^ cfu g^−1^) in 8/10 birds inoculated with 11168-O MOMP^268T/G^. Similarly, levels of 11168-O MOMP^268T/G^ mutant in the liver were below the limit of detection in 10/10 birds 7 days post-infection.

Loss of *O-*glycans, for example PseAm, from *C. jejuni* flagellins has previously been shown to affect the ability of the organism to auto-aggregate, form biofilms, adhere to and invade monolayers of INT407 cells and cause disease in *in vivo* models of infection [19]. We therefore hypothesized that loss of the MOMP *O-*glycan may also lead to similar effects. As expected, 11168MOMP^268T/G^ had a significant reduced propensity to auto-agglutinate or form biofilms compared with wild-type (figure 8*a* and *b*, respectively). Furthermore, the addition of soluble BgAgs to the culture media led to reduced biofilm formation by NCTC11168, suggesting that BgAgs compete with biofilm determinants on flagellin and MOMP (see electronic supplementary material, figure S3). Additionally, pre-treatment of Caco-2 cells with purified glycosylated MOMP (but not MOMP^268T/G^) significantly inhibited the association of wild-type NCTC11168 to Caco-2 cells (figure 8*c*), which express H-I, H-II and Le^b^ BgAgs [40]. Adherence of 11168MOMP^268T/G^ to Caco-2 cells was significantly reduced compared with wild-type NCTC11168. Levels of 11168MOMP^268T/G^ adhesion were unaffected by the pre-treatment of Caco-2 cells with either MOMP derivative (figure 8*c*).

Finally, the ability of 11168MOMP^268T/G^ to colonize chickens was determined. To do this, six-week-old birds (*n* = 10 per group) were challenged with 3 × 10^3^ cfu wild-type strain NCTC11168-O (a robust colonizing strain, derived from NCTC11168 by passage through chicken) or its 11168-O MOMP^268T/G^ mutant by oral gavage. Caecal colonization levels were determined in birds from each group at 7 days post-challenge. The results showed a significant reduction in the geometric mean colonization levels in the caeca and liver in the MOMP^268T/G^ group compared with the wild-type (figure 8*d*). These results confirm the importance and biological relevance of MOMP *O-*glycosylation in the establishment of colonization *in vivo*.

## 4. Discussion

There is currently a need for a better understanding of the pathogenic mechanisms underlying the infection of humans and colonization of other animals, particularly chickens, by *C. jejuni.* Here, we confirm that *C. jejuni*, like *H. pylori*, can bind to BgAgs and to the lacto series type I and II, a characteristic that is likely to contribute to the ability of this intestinal bacterium to interact with epithelial cells lining the gastrointestinal tract. The high degree of specificity exhibited by *H. pylori* in its BgAg-binding interactions [28,29] is in contrast to our findings, and those of Day *et al*. [23], showing that *C. jejuni* can bind to a wide range of related antigens. This may reflect the contrast between the very restricted host range of *H. pylori*, which infects only humans, and that of *C. jejuni*, which is able to establish infection in a wide range of birds and mammals. The latter may thus have gained an evolutionary advantage by broadening its specificity and thus maximizing its survival in different hosts. Whether *C. jejuni* can bind to non-human BgAgs, especially those expressed by chickens, remains to be experimentally confirmed, although our *in vivo* data suggest that this is a possibility since a *C. jejuni* derivative lacking the MOMP *O-*glycan (and hence exhibiting reduced BgAg-binding) showed a significantly reduced ability to colonize chickens.

Identification of both FlaA and MOMP as BgAg-binding adhesins was surprising as these proteins have little in common in terms of their primary sequence, predicted structure or known functions. FlaA is the major structural subunit of the flagellum and is thus required for motility. Intact and fully functional flagella are required for the colonization of chickens, invasion into human epithelial cells and translocation of human intestinal cell monolayers [41–43]. MOMP is a member of the trimeric bacterial porin family and forms voltage-sensitive cation-selective ion channels [44]. It is predicted to comprise outer membrane-spanning beta strands separated by short periplasmic and longer surface-exposed loops [30]. In addition to its porin functions, MOMP has been shown to bind to the surface of human epithelial cells and to the basement membrane protein fibronectin [45]. Interestingly, it shares this latter property with FlaA, as well as the outer membrane protein CadF [45]. Thus, both MOMP and FlaA appear to mediate binding to multiple host cell molecules, which now includes BgAgs. MOMP is encoded by the *porA* gene, which is extremely genetically diverse with most variation occurring in regions encoding surface loops (particularly loop 4), suggesting that immune selection strongly influences the diversity of the MOMP protein [46].

*Campylobacter* flagella and numerous surface proteins are post-translationally decorated with glycan molecules [47]. This led us to hypothesize that, like FlaA, MOMP is glycosylated, and that BgAg binding by these two diverse ligands may be influenced by the presence of surface glycans. *Campylobacter jejuni* harbours both *N*- and *O*-linked glycosylation systems [47]. The genes known to contribute to the *O*-glycosylation of *C. jejuni* flagellin are clustered in the flagellar glycosylation locus [19]. We have demonstrated that, like fla gellin, MOMP is also *O-*glycosylated, the first demonstration that a non-flagellin protein is glycosylated by this system. Our results also demonstrate that the MOMP *O-*glycan contains an epitope which mimics a GM1 ganglioside epitope: Gal(β1–3)-GalNAc(β1–4)-Gal-[Neu5Acα2–3]-(β1–4)-Glcβ1-ceramide. As MOMP is highly immunogenic, the expression of a glycosylated form of MOMP may play a hitherto underappreciated role in the development of autoimmune diseases, for example GBS. This potentially significant finding requires further experimental consideration.

Our finding that MOMP is *O-*glycosylated may provide an explanation for a recent observation made by Hitchen *et al*. [48]. They showed that the product of the phase variable gene *Cj1295* in *C. jejuni* NCTC11168 was responsible for the modification of Pse residues present on flagellins with di-*O*-methyl-glyceric acid [48]. Immunoblots of whole-cell extracts using rabbit polyclonal anti-flagellin antibodies showed the differing electrophoretic mobilities of flagellin with or without this Pse modification; however the anti-flagellin antibodies also detected one additional protein: a 45 kDa protein, subsequently identified as MOMP [48]. The detection of MOMP using anti-flagellin was suggested to be due to non-specific binding [48]. However, in light of our data confirming the *O-*glycosylation of MOMP, we hypothesize that the cross-reactivity is due to the presence of shared epitopes, likely to be shared or similar *O-*glycan determinants. We are currently investigating possible similarities between the MOMP and flagellin *O-*glycan repertoires.

Having shown glycosylation, we identified T^268^ as the target for the *O*-glycosylation of MOMP in strain NCTC11168; this residue is within the sixth predicted surface-exposed loop, but is conserved in only 25 of the 46 sequences examined (see electronic supplementary material, figure S4). These included six clinical isolates tested for their ability to bind BgAgs: three high BgAg binders and three isolates with low BgAg-binding activity. T^268^ was conserved in all three high binders, but also in two of the three low binders. Previously, Seal *et al*. [49] reported that arginine was substituted for T^268^ in the MOMP sequence of the isolate A74/C compared with NCTC11168-PMSRU. The latter, a derivative of strain NCTC11168, was described as a poor chicken colonizing strain, while the former was described as a robust colonizer [49]. Changes in the isoelectric point of MOMP were among the differences reported between these two isolates, while the apparent molecular mass (as judged by electrophoretic mobility in SDS-PAGE gels) was indistinguishable between the two proteins. This is in contrast to our findings, where the substitution of glycine for T^268^ resulted in a distinct mobility shift that was indistinguishable from that observed in MOMP isolated from a *pseD* mutant. It is notable that the only other substitution in the A74/C MOMP sequence with respect to the NCTC11168 sequence was threonine for R^367^, a residue predicted to reside within the eighth surface-exposed loop according to the model proposed by Zhang *et al.* [30]. It is tempting to speculate that threonine at position 367 (numbering corresponding to the NCTC11168 sequence) may be an alternative site for *O*-glycosylation. Of the isolates examined, eight of the 46 (17%) harboured a threonine at this position and 30 (65%) had threonine at either amino acid 268 or 367. Whether threonine at this position or indeed at other positions within variant MOMP proteins are targets for the *O*-glycosylation system remains to be determined. All of the *C. jejuni* clinical isolates tested bound to at least some of the BgAgs, but the degree of binding was highly variable; variations in MOMP primary sequence and perhaps the presence of specific targets for the *O*-glycosylation system are likely to account for at least some of this variation. Likewise, variable *O*-glycosylation of FlaA may also account for variations in BgAg binding between strains.

It remains to be determined whether the bacterial *O-*glycans exert a direct (i.e. bacterial glycan to host glycan) or indirect effect on BgAg binding. However, based on our molecular modelling data, our favoured hypothesis is that the effect is indirect, whereby the presence of *O-*glycans results in conformational changes or stabilization of particular conformations of the bacterial ligands, which favour BgAg binding.

Identification of protein glycosylation profiles of *C. jejuni*, especially those related to outer membrane proteins, is fundamental for understanding the diverse pathogenicity of *C. jejuni* strains among different hosts. The model for *C. jejuni* interaction to Le^b^ and H-II antigens mediated by MOMP generated here substantially increases our knowledge about the protein and its interactions. The identification of BgAgs as receptors for the adhesive function of MOMP and the mapping of the binding site could inform vaccine design. Attempts to develop vaccines have met with little success, and concerns about the possible induction of autoimmunity-associated pathologies, for example GBS, are a barrier to the development of whole-cell vaccines for use in humans [50]. An alternative approach is to use epitopes that play an important role in colonization; glycosylated peptides would constitute particularly attractive targets for such an approach as they may be under selective pressure to be maintained, and neutralizing antibodies may reduce the ability of the pathogen to colonize the host. Immunization experiments with adhesins of uropathogenic *Escherichia coli* demonstrate the potential in the generation of antibodies with adhesion inhibitory properties [51]. Also, Houpt *et al.* [52] described an effective vaccine against intestinal *Entamoeba histolytica* infection using amoebic Gal/GalNAc lectin, which blocks both invasive disease and parasite transmission. Though the potential for developing vaccines against *C. jejuni* for use in humans may be limited, vaccines targeted at animal hosts of *C. jejuni*, particularly chicken, represent a promising approach to the reduction of *C. jejuni* contamination of poultry, and thus indirectly human infection. In a study by Huang *et al*. [53], vaccination of chickens with recombinant MOMP reconstituted in proteoliposomes demonstrated conformation-dependent humoral responses to MOMP, which confirms our finding that any modification of MOMP has important implications for protein conformation and may alter MOMP immunogenicity. The stereochemical fit of the molecular interaction between microbial adhesins and host receptors determines in part tissue tropism during the colonization process. The possibility of interfering with such interactions provides new approaches to the development of antimicrobial strategies at least during the early stage of colonization. Anti-adhesive receptor analogues may prove to be a viable strategy for lowering colonization levels in poultry.

In summary, this work provides key new insights into *C. jejuni* pathogenesis and, importantly, illustrates how glycosylation of outer membrane proteins dynamically furnishes *C. jejuni* with features that are of importance for the bacterial–host crosstalk during infection in the human host.

## 5. Material and methods

### 5.1. Bacterial strains and growth conditions

*Escherichia coli* strain JM109 was used as a host strain for the construction of mutagenic constructs (see electronic supplementary material, table S6). *E. coli* strains were grown in Lysogeny broth supplemented with ampicillin (100 µg ml^−1^), kanamycin (50 µg ml^−1^) or chloramphenicol (20 µg ml^−1^). *Campylobacter jejuni* NCTC11168 and most of the clinical isolates used in this study were kindly provided by Prof. J. Ketley (University of Leicester, UK; electronic supplementary material, table S2), with the remaining clinical isolates being obtained from the Department of Clinical Microbiology, Nottingham University Hospitals NHS Trust, Nottingham, UK (see electronic supplementary material, table S2). All *C. jejuni* strains were grown under microaerophilic conditions at 37°C on modified CCDA agar (Oxoid), or in Mueller–Hinton broth (Oxoid) supplemented with kanamycin (50 µg ml^−1^) and chloramphenicol (20 µg ml^−1^) where appropriate.

### 5.2. Digoxigenin labelling of bacteria

DIG-labelling was carried using the Roche DIG-NHS Protein labelling kit, used as per manufacturer's instructions. Briefly, cells were harvested from an overnight plate and washed three times in sodium carbonate buffer (142 mM NaHCO_3_, 8 mM Na_2_SO_3_, pH 9.0). Bacteria were resuspended to an optical density at 600 nm (OD_600_) of 1.0 and then labelled with 10 µg DIG-NHS at room temperature for 1 h. Cells were then washed three times in PBS with 0.05% Tween 20 (PBS-T) and resuspended in PBS containing 1% bovine serum albumin (1% BSA/PBS).

### 5.3. ELISA

BSA–BgAg conjugates were obtained from IsoSep, Tullinge, Sweden. Coupling of BgAgs to 96-well plates (NUNC Immobilizer Amino) was carried out by the addition of 100 µl BSA–BgAg (5 µg ml^−1^ unless stated otherwise) in sodium carbonate buffer to each well. Plates were incubated at room temperature for 2 h before unbound reagent was removed by washing three times in PBS-T. All wells were blocked by the addition of 100 µl 1% BSA/PBS and incubated for 2 h at room temperature. After further washing in PBS-T, 100 µl of DIG-tagged bacteria (at OD_600_ of 0.05 unless stated otherwise) were added to each well and incubated overnight at 4°C. Plates were washed three times in PBS-T before 100 µl anti-digoxigenin-POD solution (Roche Diagnostics; 1 in 5000 diluted in 1% BSA/PBS) was added and incubated for 1 h at room temperature. Plates were again vigorously washed in PBS-T and colour developed by adding 100 μl ABTS substrate (Roche). Plates were read with an ELISA reader (Biotek EL800) at an absorbance of 405 nm. Specific binding was determined by subtracting the binding of each strain to BSA (typically OD_405_ 0.07–0.09) from the binding to each BSA–BgAg conjugate. Statistical significance was determined using Student's *t*-test. Inhibition assays were carried out as above but after the removal of the blocking solution, 100 µl purified MOMP (5 µg ml^−1^ in 1% BSA/PBS) was added to each well and incubated at 4°C overnight. Alternatively, DIG-labelled *C. jejuni* were pre-incubated for 4 h with BgAgs (20 μg ml^−1^) in 1% BSA/PBS before being added to each well.

### 5.4. Re-tagging

NCTC11168 (1 ml of OD_600_ 0.25) was incubated for 5 h with BSA conjugated to Le^b^, H-II or H-I (20 µg) (or BSA alone as control) to which the Sulfo-SBED cross-linker (Pierce) had been conjugated according to the manufacturer's recommendations. Following washing in PBS-T, the photo-reactive cross-linker group was activated by 10 min exposure to ultraviolet irradiation (365 nm). Samples were then incubated under reducing conditions (50 mM dithiothreitol) for 10 min, washed with PBS-T and boiled three times for 5 min with 10 min intervals between boiling. Biotin-(re)tagged proteins were then purified with streptavidin-coated magnetic beads as previously described [28]. Extracted biotin-tagged proteins were separated by SDS-PAGE and bands digested with sequencing-grade trypsin (Promega) and analysed using a Micromass Tof-Spec E (Micromass).

### 5.5. Mutagenesis

To mutate *flaA*, a 5.58 kb fragment encompassing the *flaA* locus was amplified from *C. jejuni* NCTC11168 using primers FLAAF1 and FLAAR1 (see electronic supplementary material, table S7) using the Expand High Fidelity PCR system (Roche). The resulting amplicon was ligated into pGEM-T Easy according to the manufacturer's protocol to yield pNJO83 (see electronic supplementary material, table S6). This was subjected to inverse PCR using primers FLAAR2 and FLAAINV5 (see electronic supplementary material, table S7) resulting in an amplicon in which the *flaA* gene was deleted and containing a unique BamHI site which was used to introduce the chloramphenicol resistance cassette from pAV35 in place of the deleted gene. The resulting plasmid, pJS101, was confirmed by sequencing before being used to mutate *C. jejuni* by electro-transformation and allelic exchange as described previously [54]. To mutate *pglB*, chromosomal DNA extracted from *C. jejuni* 81–176Δ*pglB* (obtained from Prof. J. Ketley, University of Leicester, UK) was used to mutate NCTC11168 by natural transformation and allelic exchange as described previously [54]. To mutate *pseD*, a 3.9 kb fragment encompassing the *pseD* gene was amplified from NCTC11168 template DNA using primers Cj1333_F and Cj1333_R2 (see electronic supplementary material, table S7). The resulting amplicon was ligated into pGEM-T Easy to yield plasmid pKG352 (see electronic supplementary material, table S6). The *pseD* gene was deleted from this plasmid by inverse PCR amplification using primers Cj1333_M1 and Cj1333_M3 (see electronic supplementary material, table S7) and the resulting amplicon digested with BglII and self-ligated to produce plasmid pKG353 (see electronic supplementary material, table S6). Plasmid pKG353 was digested with BglII and ligated to a kanamycin resistance cassette obtained by the digestion of pJMK30 (see electronic supplementary material, table S6) with BamHI. A resulting plasmid containing the kanamycin resistance cassette in the reverse orientation to the deleted *pseD* gene was designated pKG354 (see electronic supplementary material, table S6) and used to mutate NCTC11168 as described above. To construct MOMP^268T/G^, a 2 kb region spanning *porA* was amplified from *C. jejuni* NCTC11168 using MOMPF4 and MOMPR4 (see electronic supplementary material, table S7) and cloned into pGEM-T Easy to yield pNJO76 (see electronic supplementary material, table S6). This construct was used as template for inverse PCR using MOMPF3 and MOMPR3, which introduced a unique BglII site in the intergenic region between *porA* and *dnaJ* into which the kanamycin cassette from pJMK30 could be introduced. The resulting plasmid, pNJO78, was confirmed by restriction digestion and sequencing before being used to mutate NCTC11168 by electro-transformation and allelic exchange to create 11168-Kan. pNJO78 was also used as template for the QuikChange Site-Directed mutagenesis kit (Stratagene). SDMOMP1 and SDMOMP2 primers (see electronic supplementary material, table S7) were designed to substitute the threonine at position 268. The resulting plasmid, pSD, was confirmed by sequencing before being used to mutate *C. jejuni* by electro-transformation and allelic exchange. Mutants lacking *flaA* in the MOMP^268T/G^ genetic background were created by electro-transforming this mutant with pJS101 (see electronic supplementary material, table S6). All mutants were confirmed by PCR and DNA sequencing (data not shown).

### 5.6. Purification of *Campylobacter jejuni* MOMP and generation of rabbit anti-MOMP antiserum

*Campylobacter* MOMP was purified to homogeneity according to a previously described method [38]. New Zealand White female rabbits were immunized subcutaneously three times at two-week intervals with 30 µg of purified NCTC11168 MOMP emulsified in Freud's complete (first immunization only) or incomplete adjuvant. Rabbit anti-MOMP antisera was used in immunoblotting experiments 1 : 5000 diluted in blocking buffer (1% BSA in PBS-T) and incubated for 2 h. After washing three times in PBS-T, membranes were incubated for 2 h with goat anti-rabbit IgG–alkaline phosphatase conjugate (Sigma), at a dilution of 1 : 2000 in blocking buffer. After washing with PBS-T, the blots were developed using BCIP/NBT-Blue liquid substrate (Sigma).

### 5.7. Identification of glycopeptides from purified MOMP

MOMP was purified under non-denaturing conditions and analysed by Nanoflow LC-MS/MS FT/ICR following in-gel protein digestion as previously described [34]. Briefly, the gel pieces were destained by washing three times in 25 mM NH_4_HCO_3_ in 50% CH_3_CN and once in 25 mM NH_4_HCO_3_ in 50% CH_3_OH. Gel pieces were dried in a vacuum centrifuge and incubated with digestion buffer (50 mM NH_4_HCO_3_, 10 ng µl^−1^ trypsin) at 37°C overnight. Peptides were extracted in 50% CH_3_CN/1% CH_3_COOH and the supernatant was evaporated to dryness in a vacuum centrifuge. Prior to MS analysis, the peptides were reconstituted in 0.2% HCOOH. After digestion, samples were subjected to LC-MS/MS analysis using a hybrid linear ion trap-FTICR mass spectrometer operated in data-dependent mode, automatically switching to MS/MS mode. MS-spectra were acquired in the FTICR, while MS/MS-spectra were acquired in the LTQ-trap. For each FTICR scan, the six most intense, double-, triple- and quadruple-charged, ions were sequentially fragmented in the linear trap by collision-induced dissociation. All tandem mass spectra were searched by Mascot (Matrix Science, London, UK) against the SwissProt database (release 54.0). Glycan structure and position were determined by the manual interpretation of the CID fragmentation spectra, using the Xcalibur software v. 2.0.7 (Thermo Scientific).

### 5.8. Detection of glycoproteins using lectins or anti-T antigen

Microtitre plates (NUNC Immobilizer Amino) were coated with NCTC11168 MOMP by placing 5 μg ml^−1^ glycoprotein in sodium carbonate buffer in each well. Untreated wells were used as negative controls. Plates were incubated at 37°C for 1 h, before being washed three times with PBS-T. Non-specific binding was blocked by adding 1% BSA/PBS to each well for 30 min at room temperature. Plates were then washed three times with PBS-T, before 3 μg ml^−1^ biotinylated lectins (Vector) or anti-T antigen (1 : 5000) in PBS were added and incubated for 30 min at room temperature. Following further washing with PBS-T, avidin–biotin-complex (peroxidase) was added according to the kit instructions and incubated for 30 min at room temperature following by washing three times with PBS-T. Finally, 100 µl of ABTS was applied and plates were read with an ELISA reader (Biotek EL800) at an absorbance of 405 nm.

### 5.9. Computational modelling

All molecular dynamic simulations were conducted by using the Amber (v. 10.0) suite of programs on the Linux/Intel PC cluster of TR-Grid maintained by TUBITAK (Scientific and Technologic Research Council of Turkey). We initiated our simulations using the following amino acid sequence:

MKLVKLSLVAALAAGAFSAANATPLEEAIKDVDVSGVLRYRYDTGNFDKNFVNNSNLNNSKQDHKYRAQVNFSAAIADNFKAFVQFDYNAADGGYGANGIKNDQKGLFVRQLYLTYTNEDVATSVIAGKQQLNLIWTDNAIDGLVGTGVKVVNNSIDGLTLAAFAVDSFMAAEQGADLLEHSNISTTSNQAPFKVDSVGNLYGAAAVGSYDLAGGQFNPQLWLAYWDQVAFFYAVDAAYSTTIFDGINWTLEGAYLGNSLDSELDDKTHANGNLFALKGSIEVNGWDASLGGLYYGDKEKASTVVIEDQGNLGSLLAGEEIFYTTGSRLNGDTGRNIFGYVTGGYTFNETVRVGADFVYGGTKTEAANHLGGGKKLEAVARVDYKYSPKLNFSAFYSYVNLDQGVNTNESADHSTVRLQLYKF.

The model was constructed based on the similar secondary structures exhibited by porins [30]. The core structure of MOMP was initiated by using the skeleton of the anion-selective porins from *Co. acidovorans* (1E54.pdb) and *P. aeruginosa* (2QTK.pdb) [55] as templates to build the beta-barrels. A combination of HyperChem (HyperChem Professional 7.51), Chimera (UCSF) [56] and the LEaP module as implemented in Amber were used to build the core and add the loops and turns. The initial structure was heated from 0 to 325 K with a restraint of 10 kcal mol^−1^ Å^−2^ on residues of beta-barrels to avoid the effect of conformational changes in loops on beta-barrels for a period of 200 ps in four steps, followed by simulations from 0 to 325 K for another period of 200 ps without any restraints in four steps. The system was further simulated at 300 K for a period of 8 ns. The ff99SB force field [57] was employed and solvation effects were incorporated using the Generalized Born model [58], as implemented [59] in Amber. A lower energy structure was chosen and this was glycosylated at the residue 268 with Gal(β1–3)-GalNAc(β1–4)-GalNAc(β1–4)-GalNAc-α-linked to the protein using xleap as implemented in Amber. The Glycam04 force field [60–62] was used to model the carbohydrate unit. The charge on the oxygen of the side chain of Thr was changed from −0.6761 to −0.4599 and the atom type of OS was assigned. The angle and dihedral parameters for dimethylether (CT-OS-CG) and dimethoxymethane (H2-CG-OS-CT) were used for the glycosylated angle and dihedral for the carbohydrate linkage. The system was minimized with 500 steps of the steepest descent minimization followed by 500 steps of conjugate gradient minimization and heated at 400 K for a period of 10 ps to avoid bad contacts with a restraint of 10 kcal mol^−1^ Å^−2^ on the protein backbone. The system was heated from 0 to 325 K for a period of 200 ps without any restraints, followed by a simulation at 300 K for a period of 3.5 ns. Root-mean-square deviation (RMSD) analysis for the complex system was carried out on the trajectories by the ptraj module of Amber (v. 10). Three-dimensional structures were displayed using Chimera (UCSF) [63], and RMSD graphics were generated by Xmgrace. Docking calculations were performed to accommodate the Lewis antigen (Le^b^) and H-II antigen within the cavity of the protein. Docking of the Le^b^ was carried out using Dock v. 6.0 [64]. Docking was performed with default settings to obtain a population of possible conformations and orientations for Le^b^ at the binding site. Spheres around the centre of the binding pocket were defined as the binding pocket for the docking runs. Dock v. 6.0 employs sphgen to produce spheres and because sphgen cannot handle more than 99 999 spheres, the residues forming loops were stripped off, and thus the calculations of spheres and grids were only performed with the beta-barrel forming the cavity. Then, the coordinates of the Le^b^ obtained were recorded and AM1-Bcc (Austian model with Bond and charge correction) [65] atomic partial charges and atom types of general amber force field [66] were assigned for them using antechamber as implemented in Amber. Xleap was used to accommodate the Le^b^ within the cavity of MOMP with combine command as well as to produce topology/parameter and coordinate files. The atom type of Le^b^ was changed to those described in the Glycam04 force field [60–62]. The system was minimized, followed by MD simulation at 300 K for about 6.0 ns. The same procedure was applied to the glycosylated protein.

### 5.10. Auto-agglutination assay

*Campylobacter jejuni* strains were grown overnight in broth under microaerophilic conditions at 37°C, with shaking. Cultures were filtered to remove large aggregates (5 µm pore size filters; Millipore) and adjusted to OD_600_ of 1.0. One millilitre of culture was transferred to a cuvette, left at room temperature and OD_600_ monitored every 10–20 min for up to 6 h.

### 5.11. Biofilm assay

The method carried out was adapted from [67,68]. *Campylobacter jejuni* strains were grown overnight in MH broth under microaerophilic conditions at 37°C, with shaking. The OD_600_ of those cultures was measured and used to inoculate MH broth (for inhibition studies containing 10 µg ml^−1^ of BgAgs) to get a final OD_600_ of 0.02. One hundred and fifty microlitres of broth was added to wells of polystyrene 96-well plates (Nunc) with control wells containing broth alone. The plates were incubated under the same microaerophilic conditions at 37°C with shaking for 2 days. Following incubation, biofilm formation was quantified. Briefly, liquid medium was removed, and the plate washed three times with PBS-T. Then, 150 μl of 1% crystal violet was added into each well, and the plate covered and stained for 5 min. The crystal violet was removed, and the dye on the plate was resolved by adding 200 μl of 33% glacial acetic acid. Plates were then measured using an ELISA reader at 595 nm.

### 5.12. Caco-2 association assays

Association assays were essentially carried out as previously described [10]. Briefly, 24-well tissue culture plates were seeded with Caco-2 cells in DMEM + 5% fetal calf serum + antibiotic/antimycotic solution (Invitrogen) and incubated until fully confluent and then for an additional 3–4 days until cells were showing evidence of differentiation (formation of domes and visible thickening of the monolayer). One day prior to infection, the culture medium was changed to DMEM + 2% serum (no antibiotics). For inhibition experiments, 20 µg ml^−1^ purified NCTC11168 MOMP or MOMPT^268T/G^, in DMEM + 2% serum, was added to monolayers for 3 h at 37°C under an atmosphere of 5% CO_2_. After washing unbound protein three times with PBS, 10^8^ cfu per well *C. jejuni* NCTC11168 or 11168MOMPT^268T/G^ were added and monolayers incubated for 3 h at 37°C under an atmosphere of 5% CO_2_. Total association was assayed by washing three times in PBS, followed by homogenization of monolayers in 0.5% sodium deoxycholate in PBS. Associated *C. jejuni* cells were enumerated by serial dilution, and plating of aliquots onto modified CCDA agar (Oxoid). Assays were repeated three times in duplicate and statistically significant differences assessed using Student's *t*-test.

### 5.13. Chicken colonization study

Briefly, 20 six-weeks-old White Leghorn chickens derived from specific pathogen-free (SPF) parents (Lohmann's, Germany) were randomly divided into two groups (*n* = 10 per group) and housed in separate isolators with unlimited food and water. At day one, all birds in both groups were challenged by oral gavage with 3 × 10^3^ cfu *C. jejuni* resuspended in 100 µl PBS. Necropsies were performed on all birds at 7 days post-challenge. Caeca and liver were removed aseptically to quantify colonization levels of bacteria by plating out serial dilutions of gut contents and homogenized liver onto blood agar plates containing Skirrow's antibiotics and cefoperazone (20 µg ml^−1^) [69]. The detection limit for colonization was 10^2^ cfu g^−1^ caecal content.

## Supplementary Material

A novel O-linked glycan modulates Campylobacter jejuni MOMP-mediated adhesion to human histo-blood group antigens and chicken colonisation

## Notes

Animal studies were conducted under the Animals Scientific Procedures Act (1986) and were approved by the AHVLA local ethical review committee.
