# Supplementary material for: A novel O-linked glycan modulates Campylobacter jejuni major outer membrane protein-mediated adhesion to human histo-blood group antigens and chicken colonization
Source: Open Biol. 2014 Jan 22;4(1):130202. doi: 10.1098/rsob.130202 (PMC3909276; doi:10.1098/rsob.130202)
Supplement: A novel O-linked glycan modulates Campylobacter jejuni MOMP-mediated adhesion to human histo-blood group antigens and chicken colonisation [file rsob130202supp1.docx]

**Supporting Online Materials**

**A novel *O*-linked glycan modulates *Campylobacter jejuni* MOMP-mediated adhesion to human histo-blood group antigens and chicken colonisation**

Jafar Mahdavi^1†^, Necmettin Pirinccioglu^2^, Neil J. Oldfield^1^, Elisabet Carlsohn^3^, Jeroen Stoof^1^, Akhmed Aslam^1^, Tim Self^1^, Shaun A. Cawthraw^4^, Liljana Petrovska^4^, Natalie Colborne^1^, Carina Sihlbom^3^, Thomas Borén^5^, Karl G. Wooldridge^1^ and Dlawer A.A. Ala’Aldeen^1†^

^1^School of Life Sciences, University of Nottingham, Nottingham, NG7 2RD, UK

^2^Department of Chemistry, University of Dicle, 21280 Diyarbakir, Turkey

^3^Proteomics Core Facility, Sahlgrenska Academy, University of Gothenburg, Box 413, SE-405 30 Gothenburg, Sweden

^4^Animal Health and Veterinary Laboratories Agency (Weybridge), Surrey, KT15 3NB, UK

^5^Department of Medical Biochemistry and Biophysics, Umeå University, SE-901 87 Umeå, Sweden

† To whom correspondence should be addressed.

**Jafar Mahdavi**  **Dlawer A.A. Ala’Aldeen**

Phone: +44-(0)115-8230748 Phone: +44-(0)115-8230748

Fax: +44-(0)115-8230759 Fax: +44-(0)115-8230759

E-mail: [Jafar.Mahdavi@nottingham.ac.uk](mailto:Jafar.Mahdavi@nottingham.ac.uk) E-mail: [daa@nottingham.ac.uk](mailto:daa@nottingham.ac.uk)

**Table S1.** Sugar structures of human blood group antigens

| Antigen | Sugar structure |
| --- | --- |
| Core-I | Galβ1→3GlcNAcβ1→3Galβ1-R |
| H-I | *Fuc1-2*- Galβ1→3GlcNAcβ1→3Galβ1-R |
| Le^a^ | Galβ1→3*(Fuc1-4)*GlcNAcβ1→3Galβ1-R |
| Le^b^ | *Fuc1-2*- Galβ1→3(*Fuc1-4*)GlcNAcβ1→3Galβ1-R |
| Core-II | Galβ1→4GlcNAcβ1→3Galβ1-R |
| H-II | *Fuc1-2*-Galβ1→4GlcNAcβ1→3Galβ1-R |
| Le^x^ | Galβ1→4-(*Fuc1-3*)GlcNAcβ1→3Galβ1-R |
| Le^y^ | *Fuc1-2*- Galβ1→4-(*Fuc1-3*)GlcNAcβ1→3Galβ1-R |
| A | (GalNAcβ1-3-)( *Fuc1-2*-) Galβ1→3/4-GlcNAcβ1→3Galβ1-R |
| B | (Galβ1-3-)( *Fuc1-2*-) Galβ1→3/4-GlcNAcβ1→3Galβ1-R |

GalNAc = *N*-acetylgalactosamine; Gal = galactose; Glc = glucose; Fuc = fucose. Le^a^ and Le^x^ can be sialylated.

**Table S2.** *C. jejuni* isolates used in this study

| Isolate*^a^* | LOS sub-group | Heat stable serotype | Phage type | ST | Clonal complex |
| --- | --- | --- | --- | --- | --- |
| NCTC11168 | ND | 2 | ND | 43 | ST-21 |
| 250 | ND | 31 | 1 | 257 | ST-257 |
| 252 | ND | 5 | 34 | ND | ND |
| 253 | 1B/3A | 50 | 34 | 860 | ST-828 |
| 254 | 1C | 4 | 1 | 50 | ST-21 |
| 255 | 2A | 12 | 2 | 21 | ST-21 |
| 257 | 3A | 50 | 5 | 257 | ST-257 |
| 258 | 2B | 5 | 1 | 205 | ST-48 |
| 259 | 3A | 19 | 33 | 257 | ST-257 |
| 260 | 1C | 37 | 1 | 21 | ST-21 |
| 262 | 2B/3A | 21 | 44 | 267 | ST-283 |
| 263 | 1C | 2 | 36 | ND | ST-45 |
| 264 | ND | 60 | 2 | 53 | ST-21 |
| 265 | 2B | 21 | 1 | 137 | ST-45 |
| 266 | 1B | 50 | 34 | 48 | ST-48 |
| 267 | 1C | 4 | 1 | 21 | ST-21 |
| 268 | 1B | 50 | 6 | 61 | ST-61 |
| 270 | 1C | 13 | 33 | 21 | ST-21 |
| 271 | 1B/1C/2A | 5 | 34 | ND | ND |
| 272 | 1C | 2 | 33 | ND | ST-21 |
| 274 | 2A | 13 | 1 | 5 | ST-353 |
| 276 | 1C | 37 | 1 | 51 | ST-443 |
| 277 | 1B/2A | 4 | 34 | 205 | ST-48 |
| 278 | ND | ND | ND | ND | ND |
| 279 | 2A | 5 | 1 | 583 | ST-45 |
| 280 | ND | 11 | 1 | 5 | ST-353 |
| 281 | ND | 31 | 39 | 257 | ST-257 |
| 282 | 1C/2A | 37 | 1 | 5 | ST-353 |
| 283 | 2A | 12 | 2 | 45 | ST-45 |
| 284 | ND | 21 | 1 | 137 | ST-45 |
| 286 | ND | 18 | 2 | 354 | ST-354 |
| 288 | 1B | 11 | 1 | 257 | ST-257 |
| 289 | 1C/2A | 6 | 39 | 267 | ST-283 |
| 290 | 1C | 13 | 1 | 21 | ST-21 |
| 291 | 1B | 50 | 34 | ND | ST-48 |
| 292 | 2A | 6 | 2 | 583 | ST-45 |
| 293 | 1A | 19 | 2 | 22 | ST-22 |
| 294 | 3A | 31 | 1 | 257 | ST-257 |
| 295 | ND | 18 | 2 | 354 | ST-354 |
| 296 | 1B | 50 | 5 | 48 | ST-48 |
| Nb1 | ND | ND | ND | ND | ND |
| Nb2 | ND | ND | ND | ND | ND |
| Nb3 | ND | ND | ND | ND | ND |
| Hb1 | ND | ND | ND | ND | ND |
| Hb2 | ND | ND | ND | ND | ND |
| Hb3 | ND | ND | ND | ND | ND |

*^a^* Strains and typing information were kindly provided by Prof. J. Ketley (University of Leicester, UK), with the exception of Nb1, Nb2, Nb3, Hb1, Hb2 and Hb3 which were provided by the Dept. of Clinical Microbiology, Nottingham University Hospitals NHS Trust, UK. ND = not determined.

**Table S3.** *C. jejuni* clinical isolates bind to a range of BgAgs

A range of DIG-labelled *C. jejuni* clinical isolates were examined to assess their ability to bind to BSA-conjugated BgAgs (10 µg ml^-1^). Specific binding was calculated by subtracting the BSA (negative control) values (typically OD_405_ 0.07-0.09) from the BSA-BgAg absorbance. Values shown are an average of triplicate samples measured at 405 nm and indicate the signal due to specific bacterial interaction with the BgAgs. The strain NCTC11168 and its mutant derivatives (11168Δ*flaA*, 11168MOMP^268T/G^ and 11168MOMP^268T/G^ Δ*flaA*) were used as controls. Clinical isolates of *C. jejuni* bound to many of the BgAgs, albeit to a variable degree, suggesting that BgAg binding is a widespread phenotypic trait amongst *C. jejuni* isolates.

| *C. jejuni* strain | Core-I | Core-II | Le^x^ | Le^b^ | H-I | H-II | Le^y^ |
| --- | --- | --- | --- | --- | --- | --- | --- |
| NCTC11168 | 0.670 | 0.561 | 0.611 | 0.720 | 0.610 | 0.315 | 0.762 |
| 11168Δ*flaA* | 0.428 | 0.320 | 0.436 | 0.438 | 0.290 | 0.211 | 0.572 |
| 11168MOMP^268T/G^ | 0.385 | 0.372 | 0.240 | 0.381 | 0.460 | 0.102 | 0.215 |
| 11168MOMP^268T/G^Δ*flaA* | 0.152 | 0.131 | 0.102 | 0.131 | 0.160 | 0.108 | 0.143 |
| 250 | 0.170 | 0.160 | 0.210 | 0.200 | 0.140 | 0.111 | 0.220 |
| 252 | 0.190 | 0.170 | 0.420 | 0.190 | 0.150 | 0.160 | 0.240 |
| 253 | 0.340 | 0.310 | 0.600 | 0.380 | 0.270 | 0.250 | 0.380 |
| 254 | 0.320 | 0.400 | 0.680 | 0.480 | 0.320 | 0.340 | 0.480 |
| 255 | 0.200 | 0.170 | 0.500 | 0.210 | 0.160 | 0.160 | 0.240 |
| 257 | 0.130 | 0.080 | 0.390 | 0.110 | 0.070 | 0.070 | 0.130 |
| 258 | 0.200 | 0.160 | 0.360 | 0.220 | 0.150 | 0.180 | 0.240 |
| 259 | 0.310 | 0.260 | 0.170 | 0.270 | 0.180 | 0.230 | 0.390 |
| 260 | 0.270 | 0.220 | 0.510 | 0.260 | 0.180 | 0.300 | 0.395 |
| 262 | 0.180 | 0.130 | 0.220 | 0.150 | 0.100 | 0.100 | 0.170 |
| 263 | 0.130 | 0.080 | 0.320 | 0.110 | 0.060 | 0.060 | 0.130 |
| 264 | 0.090 | 0.060 | 0.350 | 0.090 | 0.060 | 0.050 | 0.110 |
| 265 | 0.340 | 0.260 | 0.570 | 0.290 | 0.180 | 0.140 | 0.280 |
| 266 | 0.500 | 0.370 | 0.490 | 0.490 | 0.350 | 0.370 | 0.550 |
| 267 | 0.200 | 0.190 | 0.390 | 0.220 | 0.150 | 0.200 | 0.270 |
| 268 | 0.080 | 0.080 | 0.110 | 0.090 | 0.070 | 0.070 | 0.120 |
| 270 | 0.270 | 0.250 | 0.310 | 0.310 | 0.210 | 0.200 | 0.340 |
| 271 | 0.160 | 0.130 | 0.250 | 0.170 | 0.140 | 0.140 | 0.190 |
| 272 | 0.200 | 0.160 | 0.360 | 0.200 | 0.150 | 0.110 | 0.250 |
| 274 | 0.240 | 0.200 | 0.490 | 0.250 | 0.200 | 0.240 | 0.300 |
| 276 | 0.060 | 0.050 | 0.320 | 0.080 | 0.050 | 0.050 | 0.080 |
| 277 | 0.170 | 0.140 | 0.400 | 0.180 | 0.120 | 0.150 | 0.280 |
| 278 | 0.120 | 0.080 | 0.270 | 0.120 | 0.080 | 0.080 | 0.160 |
| 279 | 0.200 | 0.210 | 0.250 | 0.220 | 0.170 | 0.190 | 0.270 |
| 280 | 0.140 | 0.140 | 0.170 | 0.160 | 0.120 | 0.110 | 0.180 |
| 281 | 0.090 | 0.080 | 0.100 | 0.080 | 0.070 | 0.050 | 0.110 |
| 282 | 0.420 | 0.370 | 0.480 | 0.440 | 0.270 | 0.280 | 0.400 |
| 283 | 0.300 | 0.300 | 0.530 | 0.320 | 0.260 | 0.250 | 0.370 |
| 284 | 0.070 | 0.020 | 0.270 | 0.040 | 0.020 | 0.000 | 0.040 |
| 286 | 0.180 | 0.160 | 0.310 | 0.180 | 0.130 | 0.120 | 0.210 |
| 288 | 0.220 | 0.220 | 0.440 | 0.230 | 0.180 | 0.190 | 0.270 |
| 289 | 0.150 | 0.130 | 0.190 | 0.150 | 0.120 | 0.140 | 0.200 |
| 290 | 0.150 | 0.140 | 0.190 | 0.160 | 0.120 | 0.130 | 0.210 |
| 291 | 0.150 | 0.130 | 0.180 | 0.150 | 0.120 | 0.130 | 0.170 |
| 292 | 0.170 | 0.140 | 0.210 | 0.190 | 0.160 | 0.180 | 0.210 |
| 293 | 0.240 | 0.180 | 0.300 | 0.250 | 0.200 | 0.220 | 0.300 |
| 294 | 0.130 | 0.080 | 0.120 | 0.090 | 0.070 | 0.070 | 0.090 |
| 295 | 0.110 | 0.100 | 0.150 | 0.120 | 0.090 | 0.090 | 0.140 |
| 296 | 0.150 | 0.090 | 0.200 | 0.160 | 0.120 | 0.130 | 0.250 |
| Nb1 | 0.053 | 0.071 | 0.069 | 0.134 | 0.093 | 0.144 | 0.158 |
| Nb2 | 0.043 | 0.061 | 0.080 | 0.147 | 0.077 | 0.134 | 0.164 |
| Nb3 | 0.058 | 0.066 | 0.053 | 0.123 | 0.060 | 0.129 | 0.151 |
| Hb1 | 0.576 | 0.633 | 0.796 | 0.685 | 0.673 | 0.532 | 0.352 |
| Hb2 | 0.627 | 0.423 | 0.248 | 0.497 | 0.729 | 0.685 | 0.487 |
| Hb3 | 0.644 | 0.591 | 0.244 | 0.589 | 0.695 | 0.524 | 0.403 |

**Table S4.** Overview of the lectins used in this study

| Abbreviation | Names | Specificity |
| --- | --- | --- |
| GSL-II | *Griffonia (Bandeiraea) simplicifolia* lectin II | agalactosylated tri/tetra antennary glycans, GlcNAc |
| DSL | *Datura Stramonium* lectin | GlcNAc(β1-4)- ˃˃ GlcNAc |
| ECL | *Erythrina cristagalli* lectin | Gal(β1-4)-GlcNAc- |
| LEL | *Lycopersicon esculentum* (tomato) lectin | GlcNAc- trimer/tetramer |
| STL | *Solanum tuberosum* (potatoe) lectin | GlcNAc oligomers |
| VVA | *Vicia villosa* agglutinin | α-linked terminal GalNAc, GalNAc(α1-3)-Gal- |
| Jacalin | *Artocarpus integrifolia* | Gal(β1-3)-GalNAcα1-Ser/Thr |

**Figure S1.** Predicted structure of the NCTC11168 MOMP *O-*glycosylate group, Gal(β1-3)-GalNAc(β1-4)-GalNAc(β1-4)-GalNAcα1-Thr

**Figure S2.** Structure of the Lewis b (left) and H-II antigen (right)

**Table S5.** The binding free energy (PBTOT) calculated by MMPBSA for the complexes of non-glycosylated MOMP (MOMP) and glycosylated MOMP (Gly-MOMP).

| Energies | H-II | Le^b^ |
| --- | --- | --- |
| ELE MOMP | 44.59±24.88 | -37.42±10.72 |
| ELE Gly-MOMP | 46.19±29.59 | -39.56±17.13 |
| VDW MOMP | -3626214±11843 | -39.42±4.03 |
| VDW Gly-MOMP | -3629596±12329 | -39.78±7.91 |
| PBTOT MOMP | -3795720±12208 | -9.07±6.98 |
| PBTOT Gly-MOMP | -3799921±13007 | -15.64±7.11 |

ELE: the energy contribution of electrostatic interactions, VDW: the energy contribution of van der Waals interactions.

**Figure S3.** The effect of soluble BgAgs on *C. jejuni* biofilm formation. Comparison of the biofilm formed by *C. jejuni* NCTC11168 in the presence or absence of soluble BgAgs. A significant decrease in biofilm formation was observed between the non-treated (NT) and treated cultures suggesting that BgAgs compete with biofilm determinants on flagellin and MOMP. Error bars = mean of triplicate values on two occasions ± SED. **p*<0.05; ***p*<0.01; ****p*<0.001.

**Figure S4.** Alignment of *porA* from different *C. jejuni* isolates

CLUSTAL W (1.81) multiple sequence alignment using BLOSUM weight matrix, of *Campylobacter jejuni* major outer membrane amino acid sequences downloaded from the Uniprot Database ([**http://www.uniprot.org/**](http://www.uniprot.org/)). Also, three non-binder (Nb) and three high binder (Hb) clinical isolates were added into this series (in house sequencing). Amino acid positions refer to positions in strain NCTC11168 (P80672). Annotation with "**_*_**","**:**", "**.**" refers to identical, conserved, semi-conserved amino acid substitutions respectively.

Hb1 MKLVKLSLVAALAAGAFSAANATPLEEAIKDVDVSGVLRYRYDTGNFDKNFVN-NSNLNN 59

Q2LAB2 MKLVKLSLVAALAAGAFSAANATPLEEAIKDVDVSGVLRYRYDTGNFDKNFVN-NSNLNN 59

Q2LAB0 MKLVKLSLVAALAAGAFSAANATPLEEAIKDVDVSGVLRYRYDTGNFDKNFVN-NSNLNN 59

Hb2 MKLVKLSLVAALAASAFSAANATPLEEAIKDVDVSGVLRYRYDTGNFDKNFVN-NSNLNN 59

NB1 MKLVKLSLVAALAASAFSAANATPLEEAIKDVDVSGVLRYRYDTGNFDKNFVN-NSNLNN 59

Q9F791 MKLVKLSLVAALAASAFSAANATPLEEAIKDVDVSGVLRYRYDTGNFDKNFVN-NSNLNN 59

Hb3 MKLVKLSLVAALAASAFSAANATPLEEAIKDVDVSGVLRYRYDTGNFDKNFVN-NSNLNN 59

D3FNB0 MKLVKLSLVAALAAGAFSAANATPLEEAIKDVDVSGVLRYRYDTGNFDKNFVN-NSNLNN 59

Q2LAB1 MKLVKLSLVAALAAGAFSAANATPLEEAIKDVDVSGVLRYRYDTGNFDKNFVN-NSNLNN 59

Q2LAB8 MKLVKLSLVAALAAGAFSAANATPLEEAIKDVDVSGVLRYRYDTGNFDKNFVN-NSNLNN 59

Q2LAA5 MKLVKLSLVAALAAGAFSAANATPLEEAIKDVDVSGVLRYRYDTGNFDKNFVN-NSNLNN 59

Q2LAB6 MKLVKLSLVAALAAGAFSAANATPLEEAIKDVDVSGVLRYRYDTGNFDKNFVN-NSNLNN 59

Q2LA95 MKLVKLSLVAALAAGAFSAANATPLEEAIKDVDVSGVLRYRYDTGNFDKNFVN-NSNLNN 59

**P80672** MKLVKLSLVAALAAGAFSAANATPLEEAIKDVDVSGVLRYRYDTGNFDKNFVN-NSNLNN 59

Q2LAC5 MKLVKLSLVAALAAGAFSAANATPLEEAIKDVDVSGVLRYRYDTGNFDKNFVN-NSNLNN 59

Q2LAA2 MKLVKLSLVAALAAGAFSAANATPLEEAIKDVDVSGVLRYRYDTGNFDKNFVN-NSNLNN 59

Q2LAB7 MKLVKLSLVAALAAGAFSAANATPLEEAIKDVDVSGVLRYRYDTGNFDKNFVN-NSNLNN 59

Q2LAB9 MKLVKLSLVAALAAGAFSAANATPLEEAIKDVDVSGVLRYRYDTGNFDKNFVN-NSNLNN 59

Q2LA91 MKLVKLSLVAALAAGAFSAANATPLEEAIKDVDVSGVLRYRYDTGNFDKNFVN-NSNLNN 59

NB2 MKLVKLSLVAALAAGAFSAANATPLEEAIKDVDVSGVLRYRYDTGNFDKNFVN-NSNLNN 59

Q2LA98 MKLVKLILVAALAAGAFSAANATPLEEAIKDVDVSGVLRYRYDTGNFDKNFVN-NSNLNN 59

A3ZHA2 MKLVKLSLVAALAAGAFSAANATPLEEAIKDVDVSGVLRYRYDTGNFDKNFVN-NSNLNN 59

Q9F792 MKLVKLSLVAALAAGAFSAANATPLEEAIKDVDVSGVLRYRYDTGNFDKNFVN-NSNLNN 59

Q2LAC0 MKLVKLSLVAALAAGAFSAANATPLEEAIKDVDVSGVLRYRYDTGNFDKNFVN-NSNLNN 59

Q0GF63 MKLVKLSLVAALAAGAFSAANATPLEEAIKDVDVSGVLRYRYDTGNFDKNFVN-NSNLNN 59

Q2LAB3 MKLVKLSLVAALAAGAFSAANATPLEEAIKDVDVSGVLRYRYDTGNFDKNFVN-NSNLNN 59

Q2LA93 MKLVKLSLVAALAAGAFSAANATPLEEAIKDVDVSGVLRYRYDTGTFDKNWGTPNSNLND 60

Q2LAA0 MKLVKLSLVAALAAGAFSAANATPLEEAIKDVDVSGVLRYRYDTGTFDKNWGTPNSNLND 60

Q2LAC1 MKLVKLSLVAALAAGAFSAANATPLEEAIKDVDVSGVLRYRYDTGTFDKNWGTPNSNLND 60

Q2LAC4 MKLVKLSLVAALAAGAFSAANATPLEEAIKDVDVSGVLRYRYDTGTFDKNWGTPNSNLND 60

Q2LA94 MKLVKLSLVAALAAGAFSAANATPLEEAIKDVDVSGVLRYRYDTGTFDKNWGTPNSNLND 60

Q2LA92 MKLVKLSLVAALAAGAFSAANATPLEEAIKDVDVSGVLRYRYDTGNFDKNFLN-NSNLNN 59

Q2LAA4 MKLVKLSLVAALAAGAFSAANATPLEEAIKDVDVSGVLRYRYDTGNFDKNFIN-NSNLNN 59

Q2LA89 MKLVKLSLVAALAAGAFSAANATPLEEAIKDVDVSGVLRYRYDTGNFDKNFIN-NSNLNN 59

NB3 MKLVKLSLVAALAAGAFSAANATPLEEAIKDVDVSGVLRYRYETSN-DWSNANFGSGIS- 58

Q2LAA9 MKLVKLSLVAALAAGAFSAANATPLEEAIKDIDVSGVLRYRYETSN-DWSNANFGSGIS- 58

B5QHE5 MKLVKLSLVAALAASAFSAANATPLEEAIKDIDVSGVLRYRYESSN-PWSNANFGSGIS- 58

Q2LA96 MKLVKLSLVAALAASAFSAANATPLEEAIKDIDVSGVLRYRYESSN-PWSNANFGSGIS- 58

Q2LAB4 MKLVKLSLVAALAASAFSAANATPLEEAIKDIDVSGVLRYRYESSN-PWSNANFGSGIS- 58

Q2LA97 MKLVKLSLVAALAASAFSAANATPLEEAIKDIDVSGVLRYRYESSN-PWSNANFGSGIS- 58

Q2LAA7 MKLVKLSLVAALAAGAFSAANATPLEEAIKDIDVSGVLRYRYESSN-PWSNANFGSGIS- 58

Q9F788 MKLVKISLVAALAAGAFSAANATPLEEAIKDIDVSGVLRYRYESSN-PWSNANFGSGIS- 58

Q2LA87 MKLVKLSLVAALAAGAFSAANATPLEEAIKDIDVSGVLRYRYESSN-PWSNANFGSGIS- 58

Q2LA90 MKLVKLSLVAALAAGAFSAANATPLEEAIKDIDVSGVLRYRYESSN-PWSNANFGSGIS- 58

Q2LAA3 MKLVKLSLVAALAAGAFSAANATPLEEAIKDIDVSGVLRYRYESSN-PWSNGNYGSGIS- 58

Q0GF62 MKLVKLSLVAALAAGAFSAANATPLEEAIKDIDVSGVLRYRYDTSN-DWNNAGFGSGIS- 58

*****: *******.****************:**********::.. . .*.:.

Hb1 SKQDHKYRAQVNFSAAIADNFKAFVQFDYNAADGGY----GANGIKNDQKGLFVRQLYLT 115

Q2LAB2 SKQDHKYRAQVNFSAAIADNFKAFVQFDYNAADGGY----GANGIKNDQKGLFVRQLYLT 115

Q2LAB0 SKQDHKYRAQVNFSAAIADNFKAFVQFDYNAADGGY----GANGIKNDQKGLFVRQLYLT 115

Hb2 SKQDHKYRAQVNFSAAIADNFKAFVQFDYNAADGGY----GANGIKNDQKGLFVRQLYLT 115

NB1 SKQDHKYRAQVNFSAAIADNFKAFVQFDYNAADGGY----GANGIKNDQKGLFVRQLYLT 115

Q9F791 SKQDHKYRAQVNFSAAIADNFKAFVQFDYNAADGGY----GANGIKNDQKGLFVRQLYLT 115

Hb3 SKQDHKYRAQVNFSAAIADNFKAFVQFDYNAADGGY----GANGIKNDQKGLFVRQLYLT 115

D3FNB0 SKQNHKYRAQVNFSAAIADNFKAFIQFDYNAADGGY----GANGIKNDQKGLFVRQLYLT 115

Q2LAB1 SKQDHKYRAQVNFSAAIADNFKAFVQFDYNAADGGY----GANGIKNDQKGLFVRQLYLT 115

Q2LAB8 SKQDHKYRAQVNFSAAIADNFKAFVQFDYNAADGGY----GANGIKNDQKGLFVRQLYLT 115

Q2LAA5 SKQDHKYRAQVNFSAAIADNFKAFVQFDYNAADGGY----GANGIKNDQKGLFVRQLYLT 115

Q2LAB6 SKQDHKYRAQVNFSAAIADNFKAFVQFDYNAADGGY----GANGIKNDQKGLFVRQLYLT 115

Q2LA95 SKQDHKYRAQVNFSAAIADNFKAFVQFDYNAADGGY----GANGIKNDQKGLFVRQLYLT 115

**P80672** SKQDHKYRAQVNFSAAIADNFKAFVQFDYNAADGGY----GANGIKNDQKGLFVRQLYLT 115

Q2LAC5 NKQDHKYRAQVNFSAAIADNFKAFIQFDYNAVDGGT----GVNNVKNAEKGLFVRQLYLT 115

Q2LAA2 NKQDHKYRAQVNFSAAIADNFKAFIQFDYNAVDGGT----GVDNVTNAEKGLFVRQLYLT 115

Q2LAB7 NKQDHKYRAQVNFSAAIADDFKAFIQFDYNAVDGGT----GVDNVTNAEKGLFVRQLYLT 115

Q2LAB9 SKQDHKYRAQVNFSAAIADNFKAFIQFDYNAVDGGT----GVDNVTNTEKGLFVRQLYLT 115

Q2LA91 NKQDHKYRAQVNFSAAIADNFKAFIQFDYNAVDGGT----GVDNVTNAEKGLFVRQLYLT 115

NB2 NKQDHKYRAQVNFSAAIADNFKAFIQFDYNAVDGGT----GVDNVTNAEKGLFVRQLYLT 115

Q2LA98 NKQDHKYRAQVNFSAAIADNFKAFIQFDYNAVDGGT----GVDNVTNAEKGLFVRQLYLT 115

A3ZHA2 NKQDHKYRAQVNFGAAIADNFKAFIQFDYNAVDGGT----GVDNVTNAEKGLFVRQLYLT 115

Q9F792 NKQDHKYRAQVNFGAAIADNFKAFIQFDYNAVDGGT----GVDNVTNAEKGLFVRQLYLT 115

Q2LAC0 NKQDHKYRAQVNFGAAIADNFKAFIQFDYNAVDGGT----GVGNVKNAEKGLFVRQLYLT 115

Q0GF63 NKQDHKYRAQVNFSAAIADNFKAFIQFDYNAVDGGT----GVDNVTNAEKGLFVRQLYLT 115

Q2LAB3 NKQDHKYRAQVNFSAAIADNFKAFIQFDYNAVDGGT----GVDNATNAEKGLFVRQLYLT 115

Q2LA93 SKQDHKYRAQVNFSAAIADNFKAFIQFDYNAVDGGT----GVDNKTNAEKGLFVRQLYLT 116

Q2LAA0 SKQDHKYRAQVNFSAAIADNFKAFIQFDYNAVDGGT----GVDNKTNAEKGLFVRQLYLT 116

Q2LAC1 SKQDHKYRAQVNFSAAIADNFKAFVQFDYNAVDGGT----GVDNATNAQKGFFVRQLYLT 116

Q2LAC4 SKQDHKYRAQVNFSAAIADNFKAFVQFDYNAVDGGT----GVDNATNAQKGFFVRQLYLT 116

Q2LA94 SKQDHKYRAQVNFSAAIADNFKAFIQFDYNAVDGGT----GVDNATNAEKGLFVRQLYLT 116

Q2LA92 SKQDHKYRAQVNFSAAIADNFKAFVQFDYNAVDGGT----GVDNATNAEKGLFVRQLYLT 115

Q2LAA4 SKQDHKYRAQVNFSAAIADNFKAFVQFDYNAADGGT----GVDNATNAQKGLFVRQLYLT 115

Q2LA89 SKQDHKYRAQVNFSAAIADNFKAFVQFDYNAADGGY----GANEIKNDQKGLFVRQLYLT 115

NB3 GKQDHKYRAQVNFGAASADNFKAFVQFDYSQADGGY----GADSISNTSDTLSVRQLYLT 114

Q2LAA9 GKQDHKYRAQVNFSGAISDNFKAFVQFDYNSQDGGY----GADSISNTSDTLSVRQLYLT 114

B5QHE5 GKQDHKYRAQVNFSGAISDNFKAFVQFDYNSQDGGY----GADSISNTSDTLSVRQLYLT 114

Q2LA96 GKQDHKYRAQVNFSGAISDNFKAFVQFDYNSQDGGY----GADSISNTSDTLSVRQLYLT 114

Q2LAB4 GKQDHKYRAQVNFSGAISDNFKAFVQFDYNSQDGGY----GTDSISNTSDTLTVRQLYLT 114

Q2LA97 GKQDHKYRAQVNFSGAISDNFKAFVQFDYNSQDGGY----GTDSISNTSDTLTVRQLYLT 114

Q2LAA7 GKQDHKYRAQVNFSGAISDNFKAFVQFDYNSQDGGY----GTDSISNTSDTLTVRQLYLT 114

Q9F788 GKQDHKYRAQVNFSGAISDNFKAFVQFDYNSQDGGY----GTDSISNTSDTLTVRQLYLT 114

Q2LA87 GKQDHKYRAQVNFSGAISDNFKAFVQFDYNSQDGGY----GTDSISNTSDTLTVRQLYLT 114

Q2LA90 GKQDHKYRAQVNFSGAISDNFKAFVQFDYNSQDGGY----GADSISNTSDTLTVRQLYLT 114

Q2LAA3 GKQDHKYRAQVNFNTAIADNFKAFVQFDYNSKDGGY----GENSISNTSDTLSVRQLYLT 114

Q0GF62 GKQTHNYRAQINFSGAIADNFKAFVQFDYAAVDGGYNVTNGTGNQRNDQNSLTVRQLYLT 118

.** *:****:**. * :*:****:**** *** * . * .. : *******

Hb1 YTNEDVATSVIAGKQQLNLIWTDNAIDGLVGTGVKVVNNSIDGLTLAAFAVDSFMAAEQG 175

Q2LAB2 YTNEDVATSVIAGKQQLNLIWTDNAIDGLVGTGVKVVNNSIDGLTLAAFAVDSFMAAEQG 175

Q2LAB0 YTNEDVATSVIAGKQQLNLIWTDNAIDGLVGTGVKVVNNSIDGLTLAAFAVDSFMAAEQG 175

Hb2 YTNEDVATSVIAGKQQLNTIWTDNGVDGLVGTGIKVVNNSIDGLTLAAFAVDSFMAEEQG 175

NB1 YTNEDVATSVIAGKQQLNTIWTDNGVDGLVGTGIKVVNNSIDGLTLAAFAVDSFMAEEQG 175

Q9F791 YTNEDVATSVIAGKQQLNTIWTDNGVDGLVGTGIKVVNNSIDGLTLAAFAVDSFMAEEQG 175

Hb3 YTNEDVATSVIAGKQQLNTIWTDNGVDGLVGTGIKVANNSIDGLTLAAFAVDSFMAEEQG 175

D3FNB0 YTNEDVATSVIAGKQQLNTIWTDNGVDGLVGTGIKVVNNSIDGLTLAAFAVDSFMAEEQG 175

Q2LAB1 YTNEDVATSVIAGKQQLNTIWTDNGVDGLVGTGIKVVNNSIDGLTLAAFAVDSFMAEEQG 175

Q2LAB8 YTNEDVATSVIAGKQQLNTIWTDNGVDGLVGTGIKVVNNSIDGLTLAAFAVDSFMAEEQG 175

Q2LAA5 YTNEDVATSVIAGKQQLNTIWTDNGVDGLVGTGIKVVNNSIDGLTLAAFAVDSFMAEEQG 175

Q2LAB6 YTNEDVATSVIAGKQQLNTIWTDNGVDGLVGTGIKVVNNSIDGLTLAAFAVDSFMAEEQG 175

Q2LA95 YTNEDVATSVIAGKQQLNTIWTDNGVDGLVGTGIKVVNNSIDGLTLAAFAVDSFMAAEQG 175

**P80672** YTNEDVATSVIAGKQQLNLIWTDNAIDGLVGTGVKVVNNSIDGLTLAAFAVDSFMAAEQG 175

Q2LAC5 YTNEDVATSVIAGKQQLNLIWTDNAIDGLVGTGVKVVNNSIDGLTLAAFAADSFMAAEQG 175

Q2LAA2 YTNEDVATSVIAGKQQLNLIWTDNAIDGLVGTGVKVVNNSIDGLTLAAFAADSFMAAEQG 175

Q2LAB7 YTNEDVATSVIAGKQQLNLIWTDNAIDGLVGTGVKVVNNSIDGLTLAAFAADSFMAAEQG 175

Q2LAB9 YTNEDVATSVIAGKQQLNLIWTDNAIDGLVGTGVKVVNNSIDGLTLAAFAADSFMAAEQG 175

Q2LA91 YTNEDVATSVIAGKQQLNLIWTDNAIDGLVGTGVKVVNNSIDGLTLAAFAVDSFMAAEQG 175

NB2 YTNEDVATSVIAGKQQLNLIWTDNAIDGLVGTGIKVVNNSIDGLTLAAFAADSFMAAEQG 175

Q2LA98 YTNEDVATSVIAGKQQLNLIWTDNAIDGLVGTGIKVVNNSIDGLTLAAFAADSFMAAEQG 175

A3ZHA2 YTNEDVATSVIAGKQQLNLIWTDNAIDGLVGTGVKVVNNSIDGLTLAAFAADSFMAAEQG 175

Q9F792 YTNEDVATSVIAGKQQLNLIWTDNAIDGLVGTGVKVVNNSIDGLTLAAFAADSFMAAEQG 175

Q2LAC0 YTNEDVATSVIAGKQQLNLIWTDNAIDGLVGTGVKVVNNSIDGLTLAAFAADSFMAAEQG 175

Q0GF63 YTNEDVATSVIAGKQQLNFIWTDNAIDGLVGTGVKVVNNSIDGLTLAAFAVDSFMAAEQG 175

Q2LAB3 YTNEDVATSVIAGKQQLNLIWTDNAIDGLVGTGVKVVNNSIDGLTLAAFAVDSFMTAEQG 175

Q2LA93 YTNEDVATSVIAGKQQLNIIWTDNGVDGLVGTGIKVVNNSIDGLTLAAFAVDSFMAAEQG 176

Q2LAA0 YTNEDVATSVIAGKQQLNIIWTDNGVDGLVGTGIKVVNNSIDGLTLAAFAVDSFMAAEQG 176

Q2LAC1 YTNEDVATSVIAGKQQLNIIWTDNGIDGLVGTGVKVVNNSIDGLTLAAFAVDSFMATEQG 176

Q2LAC4 YTNEDVATSVIAGKQQLNIIWTDNGIDGLVGTGVKVVNNSIDGLTLAAFAVDSFMATEQG 176

Q2LA94 YTNEDVATSVIAGKQQLNTIWTDNGIDGLVGTGVKVVNNSIDGLTLAAFAVDSFMATEQG 176

Q2LA92 YTNEDVATSVIAGKQQLNIIWTDNGVDGLVGTGVKVVNNSIDGLTLAAFAVDSFMATEQG 175

Q2LAA4 YTNEDVATSVIAGKQQLNTIWTDNGIDGLVGTGVKVVNNSIDGLTLAAFAVDSFMAEEQG 175

Q2LA89 YTNEDVATSVIAGKQQLNTIWTDNGIDGLVGTGVKVINNSIDGLTLAAFAVDSFMAAEQG 175

NB3 YTNEDVATSVIAGKQQLNTIWTDNGIDGLVGTGIKVVNNSIDGLTLAAFAMDSFNEEVPA 174

Q2LAA9 YTNEDVATSVIAGKQQLNTIWTDNGIDGLVGTGIKVVNNSIDGLTLAAFAMDSFNEEVPA 174

B5QHE5 YTNEDVATSVIAGKQQLNTIWTDNAIDGLVGTGIKVVNNSIDGLTLAAFAMDSFNEASDT 174

Q2LA96 YTNEDVATSVIAGKQQLNTIWTDNAIDGLVGTGIKVVNNSIDGLTLAAFAMDSFNEASDT 174

Q2LAB4 YTNEDVATSVIAGKQQLNTIWTDNGIDGLVGTGVKVVNNSIDGLTLAAFAMDSFNEASDT 174

Q2LA97 YTNEDVATSVIAGKQQLNTIWTDNGVDGLVGTGIKVVNNSIDGLTLAAFAMDSFNEASDT 174

Q2LAA7 YTNEDVATSVIAGKQQLNTIWTDNGIDGLVGTGVKVVNNSIDGLTLAAFAMDSFNEASDT 174

Q9F788 YTNEDVATSVIAGKQQLNTIWTDNGIDGLVGTGVKVVNNSIDGLTLAAFAMDSFNEASDT 174

Q2LA87 YTNEDVATSVIAGKQQLNTIWTDNAIDGLVGTGVKVVNNSIDGLTLAAFAMDSFNEASDT 174

Q2LA90 YTNEDVATSVIAGKQQLNFIWTDNAIDGLVGTGIKVVNNSIDGLTLAAFAMDSFNEASDT 174

Q2LAA3 YTNEDVATSVIAGKQQLNTIWTDNGVDGLVGTGIKVVNNSIDGLTLAAFAMDSFNEASDT 174

Q0GF62 YTNEDVATSVIAGKQQLNTIWTDNDIDGLVGTGIKVVNNSIDGLTLAAFAVDSYNTDE-- 176

****************** ***** :*******:** ************* **:

Hb1 AD----------LLGHS-TTS----TTQKAAPFKVDSVGNLYGAAAVGSYDLAGGQFNPQ 220

Q2LAB2 AD----------LLGHS-TTSTTH-TTQKAAPFKVDSVGNLYGAAAVGSYDLAGGQFNPQ 223

Q2LAB0 AD----------LLGHS-TTS----TTQKAAPFKVDSVGNLYGAAAVGSYDLAGGQFNPQ 220

Hb2 AD----------LLGQS-TIS----TTQNAAPFKVDSVGNLYGAAAVGSYDLAGGQFNPQ 220

NB1 AD----------LLGQS-TIS----TTQNAAPFKVDSVGNLYGAAAVGSYDLAGGQFNPQ 220

Q9F791 AD----------LLGQS-TIS----TTQNAAPFKVDSVGNLYGAAAVGSYDLAGGQFNPQ 220

Hb3 AD----------LLGQS-TIS----TTQNAAPFKVDSVGNLYGAAAVGSYDLAGGQFNPQ 220

D3FNB0 AD----------LLGKS-TIS----TTQKAAPFQADSLGNLYGAAAVGSYDLAGGQFNPQ 220

Q2LAB1 AD----------LLGQS-TIS----TTQKAAPFQADSLGNLYGAAAVGSYDLAGGQFNPQ 220

Q2LAB8 AD----------LLGQS-TIS----TTQKAAPFQADSLGNLYGAAAVGSYDLAGGQFNPQ 220

Q2LAA5 TD----------LLGQS-TIS----TTQNTAPFQADSLGNLYGAAAVGSYDLAGGQFNPQ 220

Q2LAB6 TD----------LLGQS-TIS----TTQNTALFQADSLGNLYGAAAVGSYDLAGGQFNPQ 220

Q2LA95 AD----------LLGHSNTST----ATPNQVPFKVDSVGNLYGAAAVGSYDLAGGQFNPQ 221

**P80672** AD----------LLEHS-NIS----TTSNQAPFKVDSVGNLYGAAAVGSYDLAGGQFNPQ 220

Q2LAC5 AD----------LLGHS-NIS----TTSKQAPFKVDSVGNLYGAAAVGSYDLAGGQFNPQ 220

Q2LAA2 AD----------LLGHS-TTSTT----QATAPFKVDSVGNLYGAAAVGSYDLAGGQFNPQ 220

Q2LAB7 AD----------LLGHS-TTSTT----QATAPFKVDSVGNLYGAAAVGSYDLAGGQFNPQ 220

Q2LAB9 AD----------LLEHS-TISTT----QNAAPFKVDSVGNLYGAAAVGSYDLAGGQFNPQ 220

Q2LA91 AD----------LLGHS-NISTT---NANQAPFKVDSVGNLYGAAAVGSYDLAGGQFNPQ 221

NB2 AD----------LLGHS-NIST----TPNQAPFKVDSVGNLYGAAAVGSYDLAGGQFNPQ 220

Q2LA98 AD----------LLGHR-NISTI---TPNQAPFKVDSVGNLYGAAAVGSYDLAGGQFNPQ 221

A3ZHA2 AD----------LLGHS-NISTT---S-NQVPFKVDSVGNLYGAAAVGSYDLAGGQFNPQ 220

Q9F792 AD----------LLGHS-NISTT---S-NQAPFKVDSVGNLYGAAAVGSYDLAGGQFNPQ 220

Q2LAC0 AD----------LLGHS-NTSTA---TPNQAPFKVDSVGNLYGAAAVGSYDLAGGQFNPQ 221

Q0GF63 AE----------LLGHS-NIS----TTSNQAPFKVDSVGNLYGAAAVGSYDLAGGQFNPQ 220

Q2LAB3 AD----------LLGHN------------GSQFNPDSIGNLYGAAAVGSYDLAGGQFNPQ 213

Q2LA93 SD----------LVG------------ANN-TFKVDSIGNLYGAAAVGSYDLAGGQFNPQ 213

Q2LAA0 SD----------LVG------------ANNSTFKVDSIGNLYGAAAVGSYDLAGGQFNPQ 214

Q2LAC1 SD----------LVG------------HNGSQFNPDSIGNLYGAAAVGSYDLAGGQFNPQ 214

Q2LAC4 SD----------LVG------------HNGSQFNPDSIGNLYGAAAVGSYDLAGGQFNPQ 214

Q2LA94 SD----------LVG------------HNGSKFSPDSIGNLYGAAAVGSYDLAGGQFNPQ 214

Q2LA92 SD----------LLGQSTYVSND---KNNNDSFKLDSIGNLYGAAAVGSYDLAGGQFNPQ 222

Q2LAA4 AD----------LLGHS-NIS--S-AN-NSAPFKLDSIGNLYGGAAVGSYEFLGGQFNPQ 220

Q2LA89 AD----------LLGHS-NIS--S-AKPNIAPFKLDSIGNLYGGAAVGSYEFLGGQFNPQ 221

NB3 TT-----------TNG-FNKGNV--NGDGDVSSALDWSKNIYGAAAIGSYDLIGGQFNPQ 220

Q2LAA9 TT-----------TNGNFNKGNV--NGDGDVSSALDWSKNIYGAAAIGSYDIAGGQFNPQ 221

B5QHE5 TVTITQD-NSQKITGVQFNRGNP--KGDSDVSGALDWSKNIYGAAAIGSYDIAGGQFNPQ 231

Q2LA96 TVTITQD-NSQKITGVQFNRGNP--KGDSDVSGALDWSKNIYGATAIGSYDIAGGQFNPQ 231

Q2LAB4 TVTITQD-SNQKITGVQFNRGNP--KGDSDVSGALDWSKNIYGAAAIGSYDIAGGQFNPQ 231

Q2LA97 TVTITQN-SSQKITGVQFNRGNP--KGDGDVSGALDWSKNIYGAAAIGSYDITGGQFNPQ 231

Q2LAA7 TVTITQD-NNQKITGVQFNRGNP--KGDSDVSGALDWSKNIYGAAAIGSYDIAGGQFNPQ 231

Q9F788 TVTITQD-NNQKITGVQFNRGNP--KGDSDVSGALDWSKNIYGAAAIGSYDIAGGQFNPQ 231

Q2LA87 TVTITQD-NNQKITGVQFNRGNP--KGDSDVSGALDWSKNIYGAAAIGSYDIAGGQFNPQ 231

Q2LA90 TVTITQN-GSQKITGVQFNRGNP--KGDGDASGALDWSKNIYGAAAIGSYDLAGGQFNPQ 231

Q2LAA3 TVIITQDPSSNKITGVQFNRGNP--KGDGDVSGALDWSKNIYGAAAIGSYDIAGGQFNPQ 232

Q0GF62 -------------QGYKDNNGRPDLTYTGDASQYLTWG-NIYGAAAVGSYDLAGGQFNPQ 222

*:**.:*:***:: *******

(**Ser 262**)(**Thr 268**)

Hb1 LWLAYWDQVAFFYAVDAAYSTTIFDGINWTLEGAYLGNSLD**S**ELDDK**T**HAN--------- 271

Q2LAB2 LWLAYWDQVAFFYAVDAAYSTTIFDGINWTLEGAYLGNSLD**S**ELDDK**T**HAN--------- 274

Q2LAB0 LWLAYWDQVAFFYAVDAAYSTTIFDGINWTLEGAYLGNSLD**S**ELDDK**T**HAN--------- 271

Hb2 LWLAYWDQVAFFYAVDAAYSTTIFDGINWTLEGAYLGNSLD**S**ELDDK**T**HAN--------- 271

NB1 LWLAYWDQVAFFYAVDAAYSTTIFDGINWTLEGAYLGNSLD**S**ELDDK**T**HAN--------- 271

Q9F791 LWLAYWDQVAFFYAVDAAYSTTIFDGINWTLEGAYLGNSLD**S**ELDDK**T**HAN--------- 271

Hb3 LWLAYWDQVAFFYAVDAAYSTTIFDGINWTLEGAYLGNSLD**S**ELDDK**T**HAN--------- 271

D3FNB0 LWLAYWDQVAFFYAVDAAYSTTIFDGINWTLEGAYLGNSLD**S**ELNDK**R**HAN--------- 271

Q2LAB1 LWLAYWDQVAFFYAVDAAYSTTIFDGINWTLEGAYLGNSLD**S**ELDDK**R**HAN--------- 271

Q2LAB8 LWLAYWDQVAFFYAVDAAYSTTIFDGINWTLEGAYLGNSLD**S**ELDDK**T**HAN--------- 271

Q2LAA5 LWLAYWDQVAFFYAVDAAYSTTIFDGINWTLEGAYLGNSLD**S**ELDDK**R**HAN--------- 271

Q2LAB6 LWLAYWDQVAFFYAVDAAYSTTIFDGINWTLEGAYLGNSLD**S**ELDDK**R**HAN--------- 271

Q2LA95 LWLAYWDQVAFFYAVDAAYSTTIFDGINWTLEGAYLGNSLD**S**ELDDT**T**HAN--------- 272

**P80672** LWLAYWDQVAFFYAVDAAYSTTIFDGINWTLEGAYLGNSLD**S**ELDDK**T**HAN--------- 271

Q2LAC5 LWLAYWDQVTFFYAVDAAYSTTIFDGINWTLEGAYLGNSLD**S**ELDDK**T**HAN--------- 271

Q2LAA2 LWLAYWDQVAFFYAVDAAYSTTIFDGINWTLEGAYLGNSLD**S**ELDDK**T**HAN--------- 271

Q2LAB7 LWLAYWDQVAFFYAVDAAYSTTIFDGINWTLEGAYLGNSLD**S**ELDDK**T**HAN--------- 271

Q2LAB9 LWLAYWDQVAFFYAVDAAYSTTIFDGINWTLEGAYLGNSLD**S**ELDDK**T**HAN--------- 271

Q2LA91 LWLAYWDQVAFFYAVDAAYSTTIFDGINWTLEGAYLGNSLD**S**ELDDK**T**HAN--------- 272

NB2 LWLAYWDQVAFFYAVDAAYSTTIFDGINWTLEGAYLGNSLD**S**ELDDK**T**HAN--------- 271

Q2LA98 LWLAYWDQVAFFYAVDAAYSTTIFDGINWTLEGAYLGNSLD**S**ELDDK**T**HAN--------- 272

A3ZHA2 LWLAYWDQVAFFYAVDAAYSTTIFDGINWTLEGAYLGNSLD**S**ELDDK**T**HAN--------- 271

Q9F792 LWLAYWDQVAFFYAVDAAYSTTIFDGINWTLEGAYLGNSLD**S**ELDDQ**A**HAN--------- 271

Q2LAC0 LWLAYWDQVAFFYAVDAAYSTTIFDGINWTLEGAYLGNSLD**S**ELDDT**T**HAN--------- 272

Q0GF63 LWLAYWDQVAFFYAVDAAYSTTIFDGINWTIEGAYLGNSID**S**ELDDT**T**HTN--------- 271

Q2LAB3 LWLAYWDQVAFFYALDASYSTTIFDGINWTLEGAYLGNSVD**S**DLDST**R**YAN--------- 264

Q2LA93 LWLAYWDQVAFFYAVDAAYSTTIFDGINWTLEGAYLGNSVD**S**DLNSA**E**HAN--------- 264

Q2LAA0 LWLAYWDQVAFFYALDVSYSTTIFDGINWTLEGAYLGNSLD**S**ELNDK**T**YAN--------- 265

Q2LAC1 LWLAYWDQVAFFYALDASYSTTIFDGINWTLEGAYLGNSVD**S**DLDSA**K**YAN--------- 265

Q2LAC4 LWLAYWDQVAFFYALDASYSTTIFDGINWTLEGAYLGNSVD**S**DLDSA**R**YAN--------- 265

Q2LA94 LWLAYWDQVAFFYALDASYSTTIFDGINWTLEGAYLGNSVD**S**DLNSA**E**YAN--------- 265

Q2LA92 LWLAYWDQVAFFYAVDAAYSTTIFDGINWTLEGAYLGNSLD**S**ELDDR**T**YAN--------- 273

Q2LAA4 LWLAYWDQVAFFYAVDAAYSTTIFDGINWTLEGAYLGNSID**S**ELDKT**T**HTN--------- 271

Q2LA89 LWLAYWDQVAFFYAVDAAYSTTIFDGINWTLEGAYLGNSID**S**ELDDK**T**HTN--------- 272

NB3 LWLAYMSDNAFLYALDAAYSTTIFDGINWSIEGAYLGNSVD**N**KLKDR**L**DAA--------N 272

Q2LAA9 LWLAYMSDNAFLYALDAAYSTTIFDGINWSIEGAYLGNSVD**N**KLKDR**L**DAA--------N 273

B5QHE5 LWLAYMSDNAFLYALDATYSTTIFDGINWTIEGAYLGNSVD**N**KLKDR**L**DAA--------N 283

Q2LA96 LWLAYMSDNAFLYALDATYSTTIFDGINWTIEGAYLGNSVD**N**KLKDR**L**DAA--------N 283

Q2LAB4 LWLAYMSDNAFLYALDAAYSTTIFDGINWTIEGAYLGNSVD**N**KLKDR**L**DAA--------N 283

Q2LA97 LWLAYMSDNAFLYALDAAYSTTIFDGINWSIEGAYLGNSVD**N**KLKDR**L**GVA--------N 283

Q2LAA7 LWLAYMSDNAFLYALDAAYSTTIFDGINWTIEGAYLGNSVD**N**KLKDR**L**DAA--------N 283

Q9F788 LWLAYMSDNAFLYALDAAYSTTIFDGINWTIEGAYLGNSVD**N**KLKDR**L**DAA--------N 283

Q2LA87 LWLAYMSDNAFLYALDAAYSTTIFNGINWTIEGAYLGNSVD**N**KLKDR**L**DAA--------N 283

Q2LA90 LWLAYMSDNAFLYALDAAYSTTIFDGINWTIEGAYLGNSVD**N**KLKDR**L**NVA--------N 283

Q2LAA3 LWLAYMSDNAFLYALDAAYNTTIFDGINWTIEGAYLGNSVD**N**KLKDR**L**DAA--------N 284

Q0GF62 LWLAYMSDNAFLYALDLAYNTTIFDGINWSIEGAYLGNSVD**N**KLKDR**F**HAAGDPESSAAN 282

***** .: :*:**:* :*.****:****::********:***.**.*.. .

(**Lys 278**)

Hb1 GNLFAL**K**GSIEVNGWDASLGGLYYGDKEKASTVVIEDQGNLGSLLAGEEIFYTTGSRLNG 331

Q2LAB2 GNLFAL**K**GSIEVNGWDASLGGLYYGDKEKASTVVIEDQGNLGSLLAGEEIFYTTGSRLNG 334

Q2LAB0 GNLFAL**K**GSIEVNGWDASLGGLYYGDKEKASTVVIEDQGNLGSLLAGEEIFYTTGSRLNG 331

Hb2 GNLFAL**K**GSIEVNGWDASLGGLYYGDKEKASTVVIEDQGNLGSLLAGEEIFYTTGSRLNG 331

NB1 GNLFAL**K**GSIEVNGWDASLGGLYYGDKEKASTVVIEDQGNLGSLLAGEEIFYTTGSRLNG 331

Q9F791 GNLFAL**K**GSIEVNGWDASLGGLYYGDKEKASTVVIEDQGNLGSLLAGEEIFYTTGSRLNG 331

Hb3 GNLFAL**K**GSIEVNGWDASLGGLYYGDKEKASTVVIEDQGNLGSLLAGEEIFYTTGSRLNG 331

D3FNB0 GNLFAL**K**GSIEVNGWDASLGGLYYGDKEKASTVVIEDQGNLGSLLAGEEIFYTTGSRLNG 331

Q2LAB1 GNLFAL**K**GSIEVNGWDASLGGLYYGDKEKASTVVIEDQGNLGSLLAGEEIFYTTGSRLNG 331

Q2LAB8 GNLFAL**K**GTIEVNGWDASLGGLYYGDKEKASTVVIEDQGNLGSLLAGEEIFYTTGSRLNG 331

Q2LAA5 GNLFAL**K**GSIEVNGWDASLGGLYYGDKEKASTVVIEDQGNLGSLLAGEEIFYTTGSRLNG 331

Q2LAB6 GNLFAL**K**GSIEVNGWDASLGGLYYGDKEKASTVVIEDQGNLGSLLAGEEIFYTTGSRLNG 331

Q2LA95 GNLFAL**K**GSIEVNGWDASLGGLYYGDKEKASTVVIEDQGNLGSLLAGEEIFYTTGSRLNG 332

**P80672** GNLFAL**K**GSIEVNGWDASLGGLYYGDKEKASTVVIEDQGNLGSLLAGEEIFYTTGSRLNG 331

Q2LAC5 GNLFAL**K**GTIEVNGWDASLGGLYYGDKEKASTVVIEDQGNLGSLLAGEEIFYTTGSRLNG 331

Q2LAA2 GNLFAL**K**GSIEVNGWDASLGGLYYGDKEKASTVVIEDQGNLGSLLAGEEIFYTTGSRLNG 331

Q2LAB7 GNLFAL**K**GSIEVNGWDASLGGLYYGDKEKASTVVIEDQGNLGSLLAGEEIFYTTGSRLNG 331

Q2LAB9 GNLFAL**K**GSIEVNGWDASLGGLYYGDKEKASTVVIEDQGNLGSLLAGEEIFYTTGSRLNG 331

Q2LA91 GNLFAL**K**GSIEVNGWDASLGGLYYGDKEKASTVVIEDQGNLGSLLAGEEIFYTTGSRLNG 332

NB2 GNLFAL**K**GSIEVNGWDASLGGLYYGDKEKASTVVIEDQGNLGSLLAGEEIFYTTGSRLNG 331

Q2LA98 GNLFAL**K**GSIEVNGWDASLGGLYYGDKEKASTVVIEDQGNLGSLLAGEEIFYTTGSRLNG 332

A3ZHA2 GNLFAL**K**GSIEVNGWDASLGGLYYGDKEKASTVVIEDQGNLGSLLAGEEIFYTTGSRLNG 331

Q9F792 GNLFAL**K**GSIEVNGWDASLGGLYYGDKEKASTVVIEDQGNLGSLLAGEEIFYTTGSRLNG 331

Q2LAC0 GNLFAL**K**GSIEVNGWDASLGGLYYGDKEKASTVVIEDQGNLGSLLAGEEIFYTTGSRLNG 332

Q0GF63 GNFFAL**K**GGIEVNGWDASLGGLYYGDKEKASTVVIEDQGNLSSLLAGEEIFYTTGSRLNG 331

Q2LAB3 GNFFAL**K**GGIEVNGWDASLGGLYYGDKEKASTVIIDDQGNLSSLLAGEEIFYTTGSRLNG 324

Q2LA93 GNLFAL**K**GSIEVNGWDASLGGLYYGDKEKASTVVIEDQGNLGSLLAGEEIFYTTGSRLNG 324

Q2LAA0 GNLFAL**K**GSIEVNGWDASLGGLYYGDKEKASTVAIEDQGNLGSLLAGEEIFYTTGSRLNG 325

Q2LAC1 GNLFAL**K**GSIEVNGWDASLGGLYYGDKEKASTVVIEDQGNLGSLLAGEEIFYTTGSRLNG 325

Q2LAC4 GNLFAL**K**GSIEVNGWDASLGGLYYGDKEKASTVVIEDQGNLGSLLAGEEIFYTTGSRLNG 325

Q2LA94 GNLFAL**K**GSIEVNGWDASLGGLYYGDKEKASTVVIEDQGNLGSLLAGEEIFYTTGSRLNG 325

Q2LA92 GNLFAL**K**GSIEVNGWDASLGGLYYGDKEKASTVVIEDQGNLGSLLAGEEIFYTTGSRLNG 333

Q2LAA4 GNLFAL**R**GSVELNGWDASLGGLYYGDKEKASTVVIEDQGNIGSLLAGEEIFYTTGSRLNG 331

Q2LA89 GNLFAL**R**GSVELNGWDASLGGLYYGDKEKASTVVIEDQGNIGSLLAGEEIFYTTGSRLNG 332

NB3 GNFFAL**R**GTVEVNGWDASLGGLYYGKKDKATVTTIEDQGNIGSLLAGEEIFYTRGSNLNG 332

Q2LAA9 GNFFAL**R**GTVEVNGWDASLGGLYYGKKDKATVTTIEDQGNIGSLLAGEEIFYTRGSNLNG 333

B5QHE5 GNFFAL**R**GTVEVNGWDASLGGLYYGKKDKITVTTIEDQGNLGSLLAGEEIFYTRGSNLNG 343

Q2LA96 GNFFAL**R**GTVEVNGWDASLGGLYYGKKDKITVTTIEDQGNLGSLLAGEEIFYTRGSNLNG 343

Q2LAB4 GNFFAL**R**GTVEVNGWDASLGGLYYGKKDKITVTTIEDQGNLGSLLAGEEIFYTRGSNLNG 343

Q2LA97 GNFFAL**R**GTVEVNGWDASLGGLYYGKKDKVTVTTIEDQGNLGSLLAGEEIFYTRGSNLNG 343

Q2LAA7 GNFFAL**R**GTVEVNGWDASLGGLYYGKKDKVTLTTIEDQGNLGSLLAGEEIFYTNGSNLNG 343

Q9F788 GNFFAL**R**GTVEVNGWDASLGGLYYGKKDKVTLTTIEDQGNLGSLLAGEEIFYTNGSNLNG 343

Q2LA87 GNFFAL**R**GTVEVNGWDASLGGLYYGKKDKVTLTTIEDQGNLGSLLAGEEIFYTNGSNLNG 343

Q2LA90 GNFFAL**R**GTVEVNGWDATLGGLYYGDKDNLTVTTIEDQGNLGSLLAGEEIFYTRGSNLNG 343

Q2LAA3 GNFFAL**R**GTVEVNGWDASLGGLYYGKKDKATVTTIEDQGNLGSLLAGQEIFYTRGSNLNG 344

Q0GF62 GNFFAL**R**GTVEVNGWDASLGGLYYGKKDKFTVTTIEDQGNLGSLLAGEEIFYTHGSRLNG 342

**:*****:*** :*:*****:*******.*:: : . *:****:.*****:***** **.***

(**Arg 352**) (**Arg 381**)

Hb1 DTGRNIFGYVTGGYTFNETV**R**VGADFVYGGTKTEAAN-HLGGGKKLEAVA**R**VDYKYSPKL 390

Q2LAB2 DTGRNIFGYVTGGYTFNETV**R**VGADFVYGGTKTEAAN-HLGGGKKLEAVA**R**VDYKYSPKL 393

Q2LAB0 DTGRNIFGYVTGGYTFNETV**R**VGADFVYGGTKTEAAN-HLGGGKKLEAVA**R**VDYKYSPKL 390

Hb2 DTGRNIFGYVTGGYTFNETV**R**VGADFVYGGTKTEAAN-HLGGGKKLEAVA**R**VDYKYSPKL 390

NB1 DTGRNIFGYVTGGYTFNETV**R**VGADFVYGGTKTEAAN-HLGGGKKLEAVA**R**VDYKYSPKL 390

Q9F791 DTGRNIFGYVTGGYTFNETV**R**VGADFVYGGTKTEAAN-HLGGGKKLEAVA**R**VDYKYSPKL 390

Hb3 DTGRNIFGYVTGGYTFNEIV**R**VGADFVYGGTKTEAAN-HLGGGKKLEAVA**R**VDYKYSPKL 390

D3FNB0 DTGRNIFGYVTGGYTFNETV**R**VGADFVYGGTKTEATN-HLGGGKKLEAVA**R**VDYKYSPKL 390

Q2LAB1 DTGRNIFGYVTGGYTFNETV**R**VGADFVYGGTKTEATN-HLGGGKKLEAVA**R**VDYKYSPKL 390

Q2LAB8 DTGRNIFGYVTGGYTFNETV**R**VGADFVYGGTKTEATN-HLGGGKKLEAVA**R**VDYKYSPKL 390

Q2LAA5 DTGRNIFGYVTGGYTFNETV**R**VGADFVYGGTKTEATN-HLGGGKKLEAVA**R**VDYKYSPKL 390

Q2LAB6 DTGRNIFGYVTGGYTFNETV**R**VGADFVYGGTKTEATN-HLGGGKKLEAVA**R**VDYKYSPKL 390

Q2LA95 DTGRNIFGYVTGGYTFNETV**R**VGADFVYGGTKTEAAN-HLGGGKKLEAVA**R**VDYKYSPKL 391

**P80672** DTGRNIFGYVTGGYTFNETV**R**VGADFVYGGTKTEAAN-HLGGGKKLEAVA**R**VDYKYSPKL 390

Q2LAC5 DTGRNIFGYVTGGYTFNETV**R**VGADFVYGGTKTEAAANHLGGGKKLEAVA**R**VDYKYSPKL 391

Q2LAA2 DTGRNIFGYVTGGYTFNETV**R**VGADFVYGGTKTEAAN-HLGGGKKLEAVA**R**VDYKYSPKL 390

Q2LAB7 DTGRNIFGYVTGGYTFNETV**R**VGADFVYGGTKTEAAN-HLGGGKKLEAVA**R**VDYKYSPKL 390

Q2LAB9 DTGRNIFGYVTGGYTFNETV**R**VGADFVYGGTKTEAAN-HLGGGKKLEAVA**R**VDYKYSPKL 390

Q2LA91 DTGRNIFGYVTGGYTFNETV**R**VGADFVYGGTKTEAAN-HLGGGKKLEAVA**R**VDYKYSPKL 391

NB2 DTGRNIFGYVTGGYTFNETV**R**VGADFVYGGTKTEDTA-HVGGGKKLEAVA**R**VNYKYSPKL 390

Q2LA98 DTGRNIFGYVTGGYTFNETV**R**VGADFVYGGTKTEDTA-HVGGGKKLEAVA**R**VDYKYSPKL 391

A3ZHA2 DTGRNIFGYVTGGYTFNETV**R**VGADFVYGGTKTEAAN-HLGGGKKLEAVA**R**VDYKYSPKL 390

Q9F792 DTGRNIFGYVTGGYTFNETV**R**VGADFVYGGTKTEAAN-HLGGGKKLEAVA**R**VDYKYSPKL 390

Q2LAC0 DTGRNIFGYVTGGYTFNETV**R**VGADFVYGGTKTEAAS-HLGGGKKLEAVA**R**VDYKYSPKL 391

Q0GF63 DTGRNIFGYVTGGYTFNETV**R**VGADFVYGGTKTEAAS-HLGGGKKLEAVA**R**VDYKYSPKL 390

Q2LAB3 DTGRNIFGYVTGGYTFNETV**R**VGADFVYGGTKTEAAN-HLGGGKKLEAVA**R**VDYKYSPKL 383

Q2LA93 DTGRNIFGYVTGGYTFNETV**R**VGADFVYGGTKTEAAS-HLGGGKKLEAVA**R**VDYKYSPKL 383

Q2LAA0 DTGRNIFGYVTGGYTFNETV**R**VGADFVYGGTKTEAAG-HLGGGKKLEAVA**R**VDYKYSPKL 384

Q2LAC1 DTGRNIFGYVTGGYTFNETV**R**VGADFVYGGTKTEAAN-HLGGGKKLEAVA**R**VDYKYSPKL 384

Q2LAC4 DTGRNIFGYVTGGYTFNETV**R**VGADFVYGGTKTEAAN-HLGGGKKLEAVA**R**VDYKYSPKL 384

Q2LA94 DTGRNIFGYVTGGYTFNETV**R**VGADFVYGGTKTEATT-HLGGGKKLEAVA**R**VDYKYSPKL 384

Q2LA92 DTGRNIFGYVTGGYTFNETV**R**VGADFVYGGTKTEAVG-HLGGGKKLEAVA**R**VDYKYSPKL 392

Q2LAA4 DTGRNIFGYVTGGYTFNETV**R**VGADFVYGGTKTETAG-HLGGGKKLEAVA**R**VDYKYSPKL 390

Q2LA89 DTGRNIFGYVTGGYTFNETV**R**VGADFVYGGTKTEAAN-HLGGGKKLEAVA**R**VDYKYSPKL 391

NB3 DIGRNIFGYVTGGYTFNETV**R**VGADFVYGGTKTNIIG---GGGKKLEAVA**R**VDYKYSPKL 389

Q2LAA9 DIGRNIFGYVTGGYTFNETV**R**VGADFVYGGTKTNIIG---QGGKKLEAVA**R**VDYKYSPKL 390

B5QHE5 DLGRNIFGYVTGGYTFNEAV**R**VGADFVYGGTKTNIIG---QGGKKLEAVA**R**VDYKYSPKL 400

Q2LA96 DLGRNIFGYVTGGYTFNEAV**R**VGADFVYGGTKTNIIG---QGGKKLEAVA**R**VDYKYSPKL 400

Q2LAB4 DLGRNIFGYVTGGYTFNEAV**R**VGADFVYGGTKTNIIG---QGGKKLEAVA**R**VDYKYSPKL 400

Q2LA97 DLGRNIFGYVTGGYTFNEAV**R**VGADFVYGGTKTNIIG---QGGKKLEAVA**R**VDYKYSPKL 400

Q2LAA7 DIGRNIFGYVTAGYTFNETV**R**VGADFVYGGTKTNIIG---QGGKKLEAVA**R**VDYKYSPKL 400

Q9F788 DIGRNIFGYVTAGYTFNETV**R**VGADFVYGGTKTNIIG---QGGKKLEAVA**R**VDYKYSPKL 400

Q2LA87 DIGRNIFGYVTAGYTFNETV**R**VGADFVYGGTKTNIIG---QGGKKLEAVA**R**VDYKYSPKL 400

Q2LA90 DLGRNIFGYVTGGYTFNEAV**R**VGADFVYGGTKTNIIG---QGGKKLEAVA**R**VDYKYSPKL 400

Q2LAA3 DLGRNIFGYVTAGYTFNEAV**R**VGADFVYGGTKTGEIG---NGGKKLEAVA**R**VDYKYSPKL 401

Q0GF62 DAGRNIFGYVTGGYTFNETV**R**VGADFVYGGTKTENVG---EGGKKLEAVA**R**VDYKYSPKL 399

* *********.****** ****************** ***************:*******

(**Ser 397**)

Hb1 NFSAFY**S**YVNLDQGVNTNESADHSTVRLQALYKF 424

Q2LAB2 NFSAFY**S**YVNLDQGVNTNESADHSTVRLQALYKF 427

Q2LAB0 NFSAFY**S**YVNLDQGVNTNESADHSTVRLQALYKF 424

Hb2 NFSAFY**S**YVNLDQGVNTNESADHSTVRLQALYKF 424

NB1 NFSAFY**S**YVNLDQGVNTNESADHSTVRLQALYKF 424

Q9F791 NFSAFY**S**YVNLDQGVNTNESADHSTVRLQALYKF 424

Hb3 NFSAFY**S**YVNLDQGVNTNESADHXTVRLQALYKF 424

D3FNB0 NFSAFY**S**YVNLDQGVNTNESADHSTVRLQALYKF 424

Q2LAB1 NFSAFY**S**YVNLDQGVNTNESADHSTVRLQALYKF 424

Q2LAB8 NFSAFY**S**YVNLDQGVNTNESADHSTVRLQALYKF 424

Q2LAA5 NFSAFY**S**YVNLDQGVNTNESADHSTVRLQALYKF 424

Q2LAB6 NFSAFY**S**YVNLDQGVNTNESADHSTVRLQALYKF 424

Q2LA95 NFSAFY**S**YVNLDQGVNTNESADHSTVRLQALYKF 425

**P80672** NFSAFY**S**YVNLDQGVNTNESADHSTVRLQALYKF 424

Q2LAC5 NFSAFY**S**YVNLDQGVNTNESADHSTVRLQALYKF 425

Q2LAA2 NFSAFY**S**YVNLDQGVNTNESADHSTVRLQALYKF 424

Q2LAB7 NFSAFY**S**YVNLDQGVNTNESADHSTVRLQALYKF 424

Q2LAB9 NFSAFY**S**YVNLDQGVNTNESADHSTVRLQALYKF 424

Q2LA91 NFSAFY**S**YVNLDQGANTNESADHSTVRLQALYKF 425

NB2 NFSAFY**S**YVNLDQGVNTNESADHSTVRLQALYKF 424

Q2LA98 NFSAFY**S**YVNLDQGVNTNESADHSTVKLQALYKF 425

A3ZHA2 NFSAFY**S**YVNLDQGVNTNESADHSTVRLQALYKF 424

Q9F792 NFSAFY**S**YVNLDQGVNTNESADHSTVRLQALYKF 424

Q2LAC0 NFSAFY**S**YVNLDQGVNTNESADHSTVRLQALYKF 425

Q0GF63 NFSAFY**S**YVNLDQGVNTNESADHSTVRLQALYKF 424

Q2LAB3 NFSAFY**S**YVNLDQGVNTNESADHSTVRLQALYKF 417

Q2LA93 NFSAFY**S**YVNLDQGVNTNESADHSTVRLQALYKF 417

Q2LAA0 NFSAFY**S**YVNLDQGVNTNESADHSTVRLQALYKF 418

Q2LAC1 NFSAFY**S**YVNLDQGVNTNESADHSTVRLQALYKF 418

Q2LAC4 NFSAFY**S**YVNLDQGVNTNESADHSTVRLQALYKF 418

Q2LA94 NFSAFY**S**YVNLDQGVNTNESADHSTVRLQALYKF 418

Q2LA92 NFSAFY**S**YVNLDQGVNTNESADHSTVRLQALYKF 426

Q2LAA4 NFSAFY**S**YVNLDEGVNTKESADHSTVRLQALYKF 424

Q2LA89 NFSAFY**S**YVNLDEGVNTKESADHSTVRLQALYKF 425

NB3 NFSAFY**S**YVNVDT---DPESTHHDAVRLQALYKF 420

Q2LAA9 NFSAFY**S**YVNVDT---DPESTHHDAVRLQALYKF 421

B5QHE5 NFSAFY**S**YVNVDT---DPESTHHDAVRLQALYKF 431

Q2LA96 NFSAFY**S**YVNVDT---DPESTHHDAVKLQALYKF 431

Q2LAB4 NFSAFY**S**YVNVDT---DPESTHHDAVRLQALYKF 431

Q2LA97 NFSAFY**S**YVNVDT---DPESTHHDAVRLQALYKF 431

Q2LAA7 NFSAFY**S**YVNVDT---DPESTHHDAVRLQALYKF 431

Q9F788 NFSAFY**S**YVNVDT---DPESTHHDAVRLQALYKF 431

Q2LA87 NFSAFY**S**YVNVDT---DPESTHHDAVRLQALYKF 431

Q2LA90 NFSAFY**S**YVNVDT---DPESTHHDAVRLQALYKF 431

Q2LAA3 NFSAFY**S**YVNVDT---DPESTHHDAVRLQALYKF 432

Q0GF62 NFSAFY**S**YVNVDR---DPESTHHDAVRLQALYKF 430

**************:* **:.* :*:*******

The major contributors in the interaction of glycosylated MOMP with Le^b^ are residues Arg^352,381^ and Lys^278^, whereas only residues 352 and 278 are involved in the interaction of non-glycosylated MOMP with Le^b^. Residues Arg^352,381^ are conserved in all sequences examined, whilst residue Lys^278^ is semi-conserved and is replaced by Arg in some strains. The molecular properties of this amino acid suggests it would be able to mediate BgAg binding through hydrogen bond formation in a similar fashion to residues Arg^352,381^.

**Table S6.** Bacterial strains and plasmids

| Strain or plasmid | Description | Source or reference |
| --- | --- | --- |
| *E. coli* strains |  |  |
| JM109 | *end*A1 *rec*A1 *gyr*A96 *thi hsd*R17 (r_K_^-^r_K_^-^) *rel*A1 *sup*E44 Δ(*lac*-*pro*AB) [F′ *tra*D36 *pro*AB *laq*I^q^ZΔM15] | Promega |
| *C. jejuni* strains |  |  |
| NCTC11168 | Wild-type strain | J. Ketley, Leicester, UK |
| 11168Δ*flaA* | *flaA* replaced with chloramphenicol cassette | This study |
| 11168Δ*pglB* | *pglB* mutant of NCTC11168 | This study |
| 11168Δ*pseD* | *pseD* mutant of NCTC11168 | This study |
| 11168-Kan | NCTC11168 with the kanamycin resistance cassette inserted downstream of *porA* | This study |
| 11168MOMP^268T/G^ | Substitution of MOMP Thr-268 to Gly | This study |
| 11168MOMP^268T/G^Δ*flaA* | *flaA* mutant of 11168MOMP^268T/G^ | This study |
| NCTC11168-O | Chicken passaged wild-type strain | This study |
| 11168-O MOMP^268T/G^ | Substitution of MOMP Thr-268 to Gly | This study |
| Plasmids |  |  |
| pGEM-T Easy | Cloning vector encoding resistance to ampicillin | Promega |
| pNJO83 | NCTC11168 *flaA* locus cloned into pGEM-T Easy | This study |
| pAV35 | Source of chloramphenicol resistance cassette | ([1](#_ENREF_1)) |
| pJS101 | pNJO83 containing the chloramphenicol resistance cassette in place of *flaA* | This study |
| pKG352 | *pseD* locus cloned into pGEM-T Easy | This study |
| pKG353 | pKG352 with *pseD* deleted by inverse PCR | This study |
| pJMK30 | Source of kanamycin resistance cassette | ([2](#_ENREF_2)) |
| pKG354 | pKG353 with kanamycin resistance cassette cloned into BglII site | This study |
| pNJO76 | 11168 *porA – dnaJ* locus cloned into pGEM-T Easy | This study |
| pNJO78 | pNJO76 containing the kanamycin resistance cassette in the intergenic region between *porA* and *dnaJ* | This study |
| pSD | pNJO78 encoding 11168MOMP^268T/G^ substitution | This study |

**Table S7.** Primers used in this study

| Primer | DNA sequence *^a^* | Restriction site |
| --- | --- | --- |
| FLAAF1 | CCTAATATCGTGCGTAGTTTAAATGC |  |
| FLAAR1 | TTTTTCTAGTAGCTTCTTTGGTGC |  |
| FLAAR2 | CGC**GGATCC**ACTTTTACTATTTAAATCAGCG | BamHI |
| FlAAINV5 | CGC**GGATCC**GGCACAGGCTAATTCTGTTC | BamHI |
| Cj1333_F | GAAGGAAATTTTACCACTCGCC |  |
| Cj1333_R2 | CTAGCTGCAAAATTTAAAGGGCGATG |  |
| Cj1333_M1 | CGC**AGATCT**GCCCCATCCATCATCTACCAC | BglII |
| Cj1333_M3 | CGC**AGATCT**GATGGAAAGGGTGAGAAGGTGAGA | BglII |
| MOMPF4 | GAGCACAAGTTAACTTCAGTGC |  |
| MOMPR4 | GAGAAGGCTTTGGCTCATCG |  |
| MOMPF3 | GGA**AGATCT**TTCAAGTCTAACTTCAAGGCG | BglII |
| MOMPR3 | GGA**AGATCT**GCTTCTTAGAATTTGTAAAGAGC | BglII |
| SDMOMP1 | CTTGATGATAAAGGACACGCTAATGGC |  |
| SDMOMP2 | GCCATTAGCGTGTCCTTTATCATCAAG |  |

*^a^* All primers were designed from the *C. jejuni* NCTC11168 genome sequence. Sequences in bold identify restriction enzyme sites. For site-directed mutagenesis, mismatched bases are underlined.

References:

1. Yao R, Alm RA, Trust TJ, Guerry P. 1993. Construction of new *Campylobacter* cloning vectors and a new mutational *cat* cassette. *Gene* **130:**127-130.

2. van Vliet AH, Wooldridge KG, Ketley JM. 1998. Iron-responsive gene regulation in a *Campylobacter jejuni fur* mutant. *J Bacteriol* **180:**5291-5298.
